# Transcriptome analysis of gene expression profiling from the deep sea in situ to the laboratory for the cold seep mussel *Gigantidas haimaensis*

**DOI:** 10.1186/s12864-022-09064-9

**Published:** 2022-12-14

**Authors:** Hua Zhang, Gaoyou Yao, Maoxian He

**Affiliations:** 1 CAS Key Laboratory of Tropical Marine Bio-Resources and Ecology, Guangdong Provincial Key Laboratory of Applied Marine Biology, South China Sea Institute of Oceanology, Chinese Academy of Sciences, Guangzhou, 510301 China; 2 Southern Marine Science and Engineering Guangdong Laboratory (Guangzhou), Guangzhou, 511458 China; 3 University of Chinese Academy of Sciences, Beijing, 100049 China; 4 Innovation Academy of South China Sea Ecology and Environmental, Engineering, Chinese Academy of Sciences, Guangzhou, 510301 China

**Keywords:** Cold seep, *Gigantidas haimaensis*, Transcriptomic analysis, Gene expression

## Abstract

**Background:**

The deep-sea mussel *Gigantidas haimaensis* is a representative species from the Haima cold seep ecosystem in the South China Sea that establishes endosymbiosis with chemotrophic bacteria. During long-term evolution, *G. haimaensis* has adapted well to the local environment of cold seeps. Until now, adaptive mechanisms responding to environmental stresses have remained poorly understood.

**Results:**

In this study, transcriptomic analysis was performed for muscle tissue of *G. haimaensis* in the in situ environment (MH) and laboratory environment for 0 h (M0), 3 h (M3) and 9 h (M9), and 187,368 transcript sequences and 22,924 annotated differentially expressed genes (DEGs) were generated. Based on Gene Ontology (GO) and Kyoto Encyclopedia of Genes and Genomes (KEGG) pathway analysis, these DEGs were enriched with a broad spectrum of biological processes and pathways, including those associated with antioxidants, apoptosis, chaperones, immunity and metabolism. Among these significantly enriched pathways, protein processing in the endoplasmic reticulum and metabolism were the most affected metabolic pathways. These results may imply that *G. haimaensis* struggles to support the life response to environmental change by changing gene expression profiles.

**Conclusion:**

The present study provides a better understanding of the biological responses and survival strategies of the mussel *G. haimaensis* from deep sea in situ to the laboratory environment.

**Supplementary Information:**

The online version contains supplementary material available at 10.1186/s12864-022-09064-9.

## Background

The cold seeps, an extreme environment on the bottom of the earth's deep sea, are characterized by flooding with hydrogen sulfide, a certain amount of heavy metals, fine-grained sediments, and hydrocarbons, mainly methane. It is widespread from shallow shelf to hadal depths and from the tropics to the poles [1, 2]. It is usually formed on the sloping seafloor of active and passive continental margins and subduction zones [3]. The water pressure is relatively high, and the water temperature usually ranges from less than 2 °C to approximately 8 °C [4]. Therefore, cold-seep animals have to adapt to harsh deep-sea environments and cope with hypoxia and rich reducing chemicals that are toxic to most animals [5–7]. Here, a food chain with chemical autotrophic bacteria as the primary producers and primary consumers such as tubeworms, snails, bivalves, sea stars, and crabs was formed [8]. Bacteria have established various symbioses with benthic animals to form an ecosystem with a unique community structure in cold seep areas [9, 10]. The bacterial symbiosis associated with mussels in the deep sea has been intensively reported in many previous studies [11–13].

Mussels, a representative of dominant species in cold seeps, such as *Bathymodiolus platifrons* [14], *Bathymodiolus childressi* [15], and *Gigantidas haimaensis* [16], form mussel beds in dense patches around seepages. During long adaptive evolution and natural selection, special metabolic mechanisms and physiological structures were formed in such extreme physical and ecological environments. Although mussels retained the capacity of filter feeding, they obtained nutrients and energy sources from bacteria such as sulfur-oxidizing bacteria, methane-oxidizing bacteria, and other symbiotic chemoautotrophic bacteria [17, 18]. In the South China Sea (SCS), more than 40 cold seep fields have been found, but only two active cold seeps, the Haima cold seeps and Formosa cold seeps, have been identified to date [1]. The Haima cold seeps were first found in 2015 and are located on the northwestern slope of the SCS [19]. Haima cold seeps have developed chemosynthetic ecosystems with living cold-seep macrofaunal communities. The mussel *G. haimaensis* is one of the most common macrofauna in the Haima cold seeps. Due to diverse cold-seep biomes in this area, Haima cold seeps provide a good opportunity to study chemoautotrophic ecosystems and environmental adaptability.

Until now, the adaptive strategies of mussels in such a heterogeneous environment have remained unclear. A recent study showed that *B. platifrons* would also gradually lose its symbionts at atmospheric pressure [20]. This process may prompt mussels to adapt to environmental fluctuations [13, 21]. Meanwhile, deep-sea mussels have been commonly used for symbiosis [22], abiotic stress [23], immune [24] and ecotoxicology [25] studies because of their significant ecological and biological characteristics, especially their ability to survive for an extended period at atmospheric pressure [26, 27]. To investigate the characteristics of the *G. haimaensis* response to environmental fluctuations, we collected mussels from the seabed and explored how long deep cold mussels can live in laboratory conditions. During this process, we collected samples regularly. According to our observations, the mussels died in the laboratory after approximately nine hours. The collected samples were used for RNA-seq and analysis. We systematically investigated genes related to antioxidative defense, detoxification, the innate immune system, and metabolism. Furthermore, we analyzed these functional genes and gene expression pattern responses to environmental fluctuations. The results extended our understanding of the adaptive mechanisms of mussels in cold seep ecosystems.

## Material and methods

### Material collection

*Gigantidas haimaensis* were collected from 1,446 m depth (Seawater temperature: 2.8 ℃; Salinity: 34.58) using the submersible ROV Haima by the cruise HYDZ6-202,005 of the Haiyang 6 research vessel of Guangzhou Marine Geological Survey. It took approximately 1 h from the seafloor to reach the surface (Seawater temperature: 30.99 ℃; Salinity: 33.67). Upon arrival at the sea surface, three mussel muscle tissues (M0) were immediately dissected and stored individually in RNAlater. Other mussels were immediately transferred to a container at surface sea water in the laboratory. After 3 h and 9 h, the muscle tissue was harvested separately (M3 and M9) and stored individually in RNAlater immediately for the subsequent isolation of RNA. Two in situ muscle samples (MH) fixed on the seafloor by a container including RNAlater were kindly provided by Pei-Yuan Qian (Hong Kong University of Science and Technology). The sample collection was approved by the South China Sea Institute of Oceanology, Chinese Academy of Sciences. All animal experiments were conducted in accordance with the relevant guidelines and regulations, in addition to the ARRIVE guidelines, and were approved by the Animal Research and Ethics Committees of the Chinese Academy of Sciences.

### RNA extraction and RNA-seq library construction

The muscle tissue was extracted for total RNA. The integrity and concentration of total RNA were examined using an Agilent Bioanalyzer 2100 and a Quawell Q5000. The RNA-seq protocol was performed following the manufacturer's instructions. Briefly, mRNA was first enriched with oligo d (T) 25 magnetic beads, and ribosomal RNA was removed. mRNA was fragmented in NEB Fragmentation Buffer. The fragmented mRNAs were reverse transcribed into first strand cDNA. Then, the second strand was synthesized according to the manufacturer's instructions. After terminal repair and ligation to sequencing adapters, purified double-stranded cDNA fragments of 250 bp ~ 300 bp were then selected, purified and subsequently PCR amplified to create the final cDNA library template for sequencing.

### Sequence assembly and unigene annotation

The adaptors with more than 10% ambiguous bases (N) and low-quality reads (more than 50% bases with Qphred <  = 20) were removed from each dataset. After that, clean and high-quality reads were de novo assembled by Trinity software [28]. The unigene gene was defined based on the longest assembled transcript of a gene. All the assembled unigenes were used as reference sequences for the muscle transcriptome.

Functional annotations of unigenes were based on the following databases: NCBI nonredundant protein sequences (Nr), NCBI nonredundant nucleotide sequences (Nt), Protein family (Pfam), Clusters of Orthologous Groups of proteins/euKaryotic Ortholog Groups (KOG/COG), a manually annotated and reviewed protein sequence database (Swiss-Prot), Kyoto Encyclopedia of Genes and Genomes (KEGG) (www.kegg.jp/kegg/kegg1.html), and Gene Ontology (GO). Nr, Nt, Swiss-Prot, and KOG were used for the alignments of unigenes by NCBI blast 2.2.28 + . hmmscan in HMMER 3.0 was used to search Pfam. Based on the Nr and Pfam annotations, Blast2GO v2.5 [29] was used to search the GO. KEGG annotations were performed by the KEGG Automatic Annotation Server (http://www.genome.jp/kegg/kaas/). The E-value threshold in the alignments to Nr, Nt, and Swiss-Prot was set to 1e – 5. The E-value thresholds in the Pfam, KOG/COG, KEGG and GO alignments were 0.01, 1e-3, 1e-10, and 1e-6, respectively.

### Identification of differentially expressed genes and functional enrichment

RSEM software [30] was used to calculate the read count of each unigene in a sample and transform it into FPKM (expected number of fragments per kilobase of transcript per million fragments mapped). The DEseq package (1.20.0) was used to perform the differential gene expression analyses. The threshold adjusted of |log2 (FoldChange)|> 2 and *p* value < 0.05. Hierarchical clustering was used to illustrate the differential gene expression patterns in different groups. GO and KEGG enrichment analyses of specific genes and DEGs were performed by Fisher's exact test with all assembled unigenes as a background and a *p* value threshold of 0.05.

### Quantitative real-time PCR (RT‒qPCR) validation of RNA-seq data

Genes with different expression patterns in RNA-seq were randomly selected to perform RT‒qPCR for validation. RNA was extracted from TRIzol reagent and subsequently reverse-transcribed using a First-strand cDNA Synthesis Kit (Toyobo, Japan) and then used to perform RT‒qPCR using SYBR green on a Roche LightCycler480 instrument (Roche, Switzerland) according to the manufacturer’s instructions. The relative quantification from the target gene was normalized to the reference gene 18S rRNA and calculated by the 2^−ΔCt^ method. All primer sequences are shown in Table S1.

### Sequence analysis

The open reading frames were predicted by ORF finder (https://www.ncbi.nlm.nih.gov/orffinder/). Phylogenetic trees were constructed by MEGA 6.0 software [31] based on the maximum likelihood method with 1000 bootstraps. Sequence alignment was performed by Clustal and CLC Main Workbench 7.7.3 software. Prediction of conserved domains was performed by MEME (http://meme-suite.org/tools/meme) and SMART (https://smart.embl.de/).

## Results

### Sequence assembly and gene annotation

**Fig. 1:**
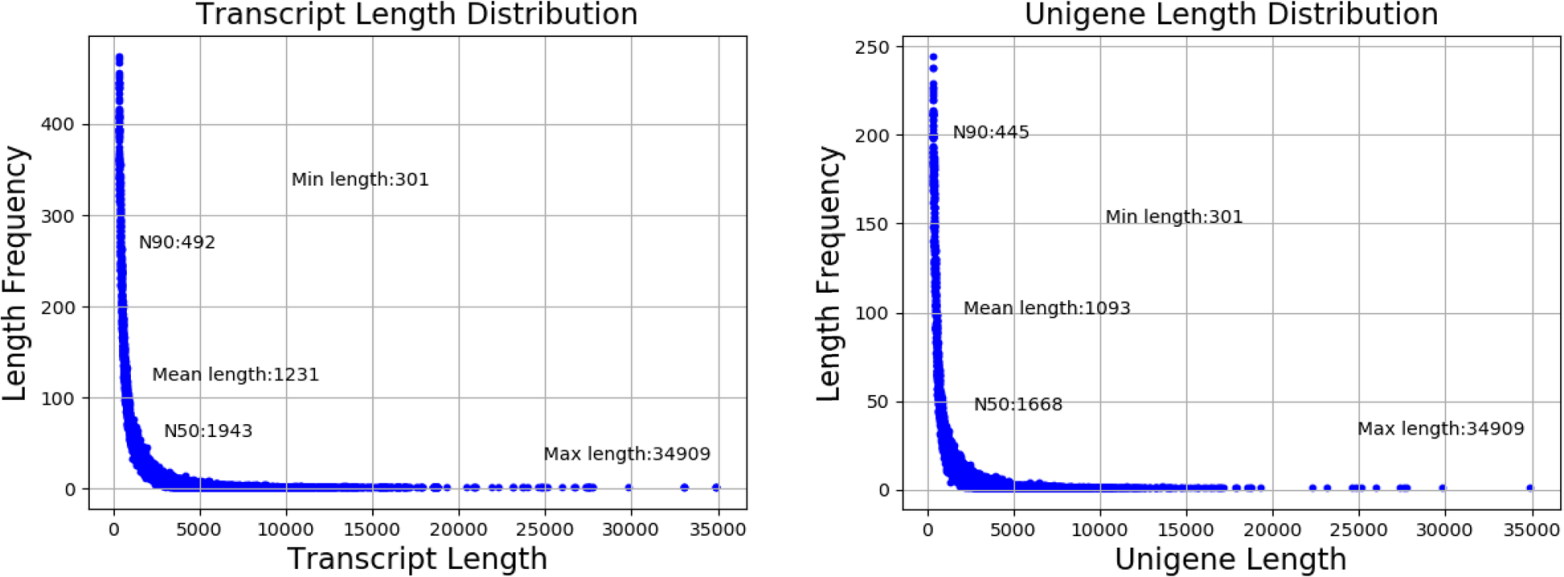
Transcript and unigene length distribution

A relatively complete and high-quality (C: 99.6 [S: 59.1%, D: 40.5%], F: 0.3%, M: 0.1%) sequencing data assembly was accomplished. A total of eleven libraries were constructed (Table S2). RNA-seq generated a total of 238,614,839 clean reads. The Q20 per sample exceeded 97%, and the Q30 was higher than 92.48%. The GC content ranged from 33.29%—38.51%. The average error rate of the sequences was 0.03% per sample. The final transcriptome generated 187,368 transcript sequences and 84,050 unigenes. The lengths of the transcripts and unigenes ranged from 301 bp to 34,909 bp (Fig. 1), with average lengths of 1,231 bp and 1,093 bp, respectively. The N50 values of transcripts and unigenes were 1,943 bp and 1,668 bp, respectively. The statistics for de novo assembly data and functional annotation results are listed in Table S3. In total, 42,169 (50.17%) transcripts were annotated in at least one database. A total of 27,733 (32.99%) transcripts were annotated to the NR database, and 25,287 (30.08%) transcripts had homologous sequences in the PFAM database. A total of 3,433 genes were commonly annotated in five databases. The best hit of most annotated transcripts (8,658) was *Mizuhopecten yessoensis* in the database, which shared 31.2% similarity (Fig. S1).

### Functional annotation of transcripts

According to the GO classification, the biological process (BP) terms were the dominant gene list, where the top 3 BP terms were cellular process, metabolic process and biological regulation; the second was molecular function (MF), where the top 3 were binding, catalytic activity and transporter activity in turn; and the third was cellular component (CC), where the top 3 were cellular anatomical entity, intracellular and protein-containing complex (Fig. S2). The enriched KEGG pathways of genes were analyzed (Fig. S3). In the term, the top 3 enriched genes were signal transduction, endocrine system and transport and catabolism. For KOG classification, the top 3 classes were signal transduction mechanisms, general function prediction only, and posttranslational modification, protein turnover, and chaperones (Fig. S4). Combined with KEGG and KOG analyses, signal transduction was the most enriched pathway.

### Differential expression analysis and GO term enrichment analysis

**Fig. 2:**
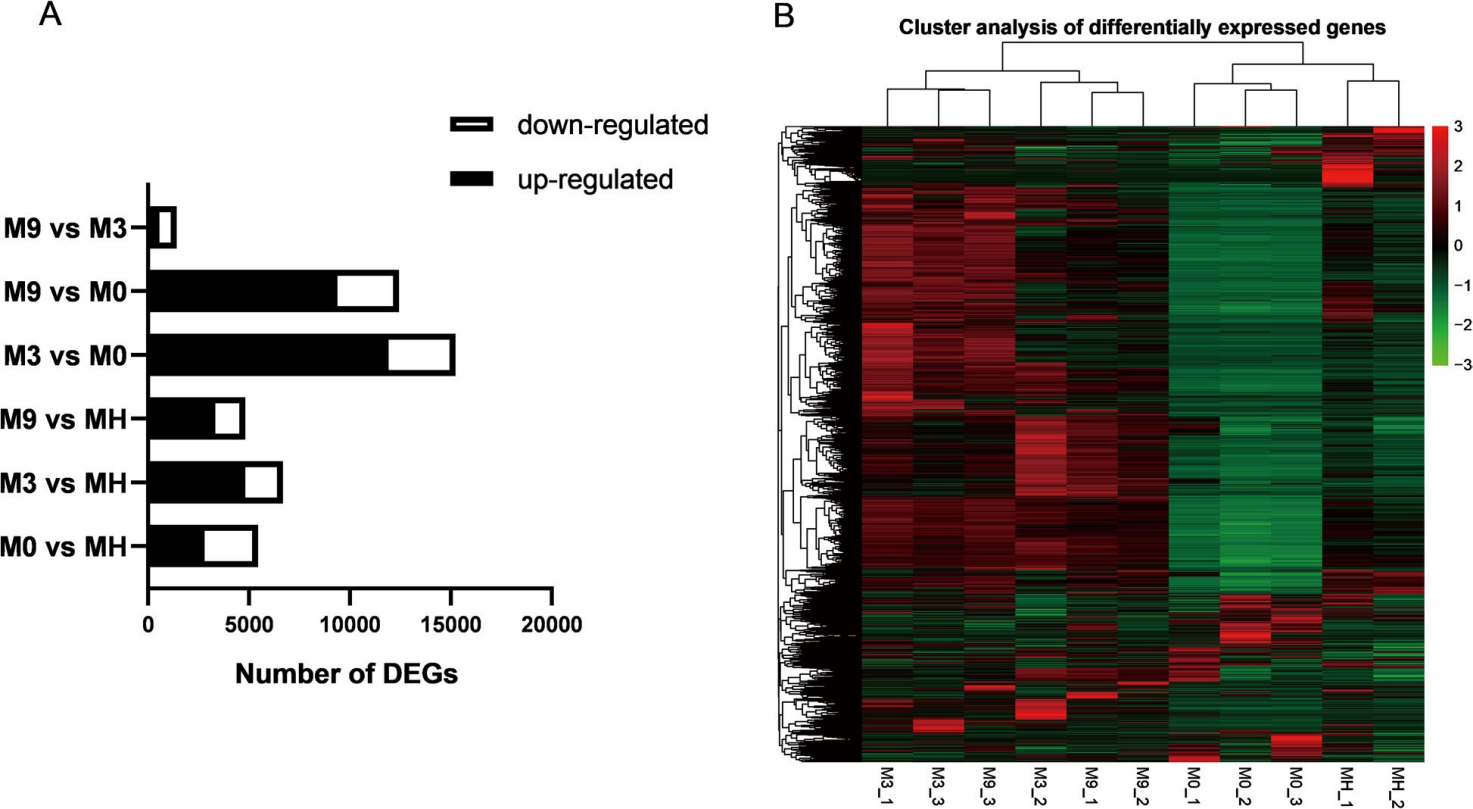
Expression analysis of DEGs. **A** Number of DEGs in comparison groups. **B** Heatmap cluster analysis of DEGs

To identify differentially expressed genes (DEGs), we then compared the gene expression profiles of four samples (Fig. 2). A total of 22,924 DEGs were annotated in databases. According to the environmental similarity, the gene expression patterns may be divided into classes: in situ (MH) and go ashore (M0, M3, and M9). In M0 vs MH, 5,460 genes showed differential expression, of which 2,800 genes were upregulated and 2,660 genes were downregulated; the most abundant GO terms were transporter activity (78) in upregulated genes and catalytic activity (774) in downregulated genes (Fig. S5A). In M3 vs MH, 6,684 genes showed differential expression, of which 4,832 genes were upregulated and 1,852 genes were downregulated; the most abundant GO terms were protein binding (668) in upregulated genes and catalytic activity (433) in downregulated genes (Fig. S5B). In M9 vs MH, 4,820 genes showed differential expression, of which 3,346 genes were significantly upregulated and 1,474 genes were significantly downregulated; the most abundant GO terms were cytoskeleton (81) in upregulated genes and catalytic activity (356) in downregulated genes (Fig. S5C). Therefore, we found that the upregulated DEGs in MH were dominantly involved in catalytic activity. In M3 vs M0, 15,249 genes showed differential expression, of which 11,921 genes were significantly upregulated and 3,328 genes were significantly downregulated; the most abundant GO terms were binding (3614) in upregulated genes and molecular function regulator (48) in downregulated genes (Fig. S5D). In M9 vs M0, 12,445 genes showed differential expression, of which 9,384 genes were significantly upregulated and 3,061 genes were significantly downregulated; the most abundant GO terms were binding (3022) in upregulated genes and transporter activity (75) and transmembrane transporter activity (75) in downregulated genes (Fig. S5E). Therefore, we found that the upregulated DEGs in M0 might be involved in transporter activity and molecular function regulation. In M9 vs M3, 1,422 genes showed differential expression, of which 569 genes were significantly upregulated and 853 genes were significantly downregulated; the most abundant GO terms were transmembrane transporter activity (41) and transporter activity (41) in upregulated genes and DNA recombination (22) in downregulated genes (Fig. S5F). In other words, the number of upregulated genes was greater than that of downregulated genes. In addition, we performed cluster analysis of the DEGs (Fig. 2). The expression levels of most DEGs in M9 and M3 were higher than those in M0 and MH.

### KEGG pathway enrichment analysis of DEGs

**Fig. 3:**
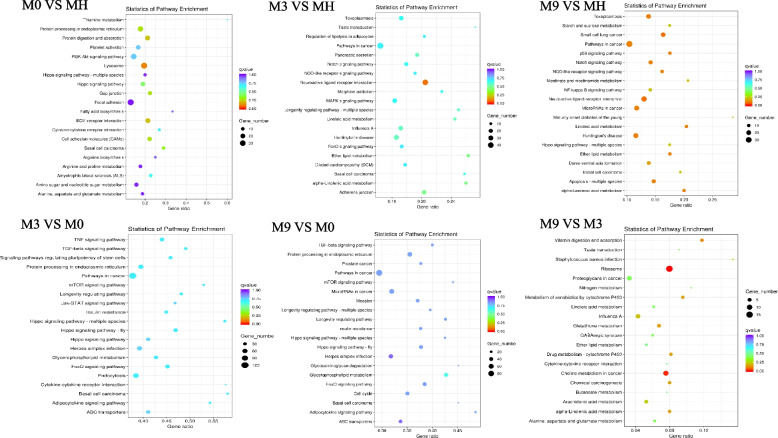
KEGG enrichment analysis of DEGs in pairwise comparison of six groups

To explore the biological processes, the DEGs were further analyzed against the KEGG database. The top 20 KEGG pathway data are shown in six groups (Fig. 3). In M0 vs MH, lysosome was the most enriched pathway. In M3 vs MH, neuroactive ligand − receptor interaction was the most enriched pathway. In M9 vs MH, neuroactive ligand‒receptor interaction and pathways in cancer were the most enriched pathways. In M3 vs M0, pathways in cancer were the most enriched pathways. In M9 vs M0, pathways in cancer were the most enriched pathways; in M9 vs M3, ribosome was the most enriched pathway. Basically, environmental information processing and metabolism were significantly enriched in the pairwise comparison of the six groups.

### Expression analysis of genes related to protein processing in the endoplasmic reticulum

**Fig. 4:**
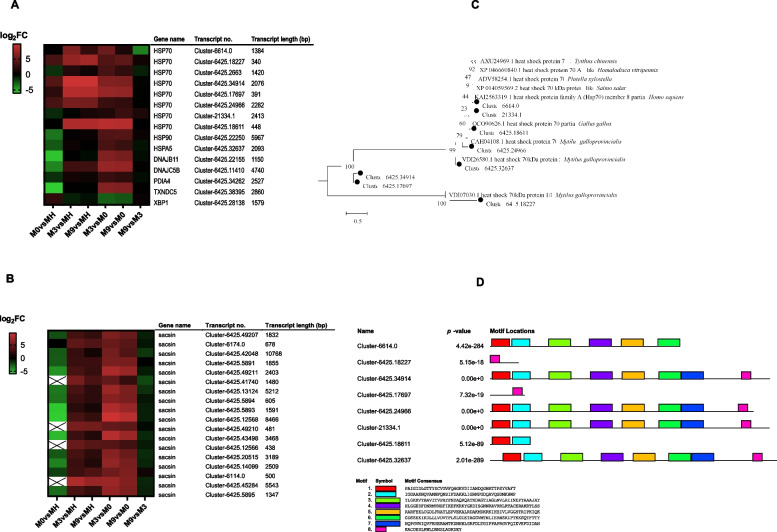
Analysis of the environmental information processing pathway genes. **A** Heatmap of DEGs related to molecular chaperones. **B** Heatmap analysis of sacsin. **C**. Phylogenetic tree of heat shock 70 kDa protein with 1000 bootstrap replications. **D** Conserved motifs of HSP70 protein in *G. haimaensis*

Environmental information processing was significantly enriched in the KEGG analysis. Several key genes involved in protein processing in the endoplasmic reticulum (ER), including the heat shock 70 kDa protein family (including HSPA5), HSP90, sacsin, dnaJ homolog subfamily C member 5 (DNAJC5), dnaJ homolog subfamily C member 5B (DNAJC5B), protein disulfide-isomerase A4 (PDIA4), thioredoxin domain-containing protein 5 (TXNDC5) and X-box binding protein 1 (XBP1), were differentially expressed in the different groups (Fig. 4A). Most of these proteins showed positive expression. Transcripts in M3 vs M0 and M9 vs M0 had similar expression trends, and fourteen transcripts showed different degrees of positive expression except for Cluster-6425.28138 (XBP1). Sacsin is also a heat shock protein. We found, compared with MH, that all eighteen transcripts of sacsin showed negative expression in M0 but presented positive expression in M3 and M9 (Fig. 4B). The HSP70 gene family was further examined. The HSP70 family contains several members and conserved HSP70 motifs. The phylogenetic analysis suggested that the HSP70 genes were mainly divided into three categories (Fig. 4C): Cluster-6425.18227 was homologous to *Mytilus galloprovincialis* HSP70-1/8; Cluster-6425.32637 was homologous to *M. galloprovincialis* HSP70-5; Cluster-6425.34914 and Cluster-6425.17697 were clearly nonhomologous to other HSP70s; Cluster-6425.24966, Cluster-6425.18611, Cluster-21334.1 and Cluster-6614.0 were homologous to *M. galloprovincialis,* insect or vertebrate HSP70s. HSP70 transcripts in *G. haimaensis* contain differentially conserved motifs. As shown in Fig. 4D, Cluster-6425.34914, Cluster-6425.24966, Cluster-21334.1, and Cluster-6425.32637 had eight differentially conserved motifs, while Cluster-6425.18227 and Cluster-6425.17697 had only one conserved motif. Cluster-6614.0 and Cluster-6425.18611 included six and two conserved motifs, respectively.

### Expression analysis of lipid metabolism-related genes

**Fig. 5:**
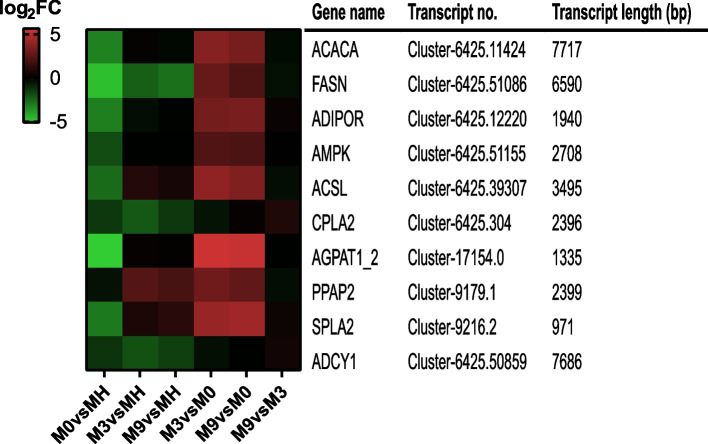
Expression analysis of the lipid metabolism pathway genes

KEGG enrichment analysis showed that lipid metabolism was significantly enriched in all pairwise comparison groups. Ten lipid metabolism-related genes, including adiponectin receptor (ADIPOR), 5'-AMP-activated protein kinase, catalytic alpha subunit (AMPK), long-chain acyl-CoA synthetase (ACSL), lysophosphatidate acyltransferase (AGPAT1_2), cytosolic phospholipase (CPLA2), phosphatidate phosphatase (PPAP2), secretory phospholipase (SPLA2), adenylate cyclase 1 (ADCY1), fatty acid synthase-like (FASN), and acetyl-CoA carboxylase-like (ACACA), were enriched in the lipid metabolism pathway. We also analyzed their transcription levels (Fig. 5). From the figure, ten transcripts had similar expression patterns in M3 vs MH, M9 vs MH, M3 vs M0 and M9 vs M0, most of which showed positive expression. However, all ten transcripts showed negative expression in M0 vs MH. Therefore, these transcripts exhibited distinctive expression patterns in one of the six pairwise comparisons.

### Expression analysis of the complement system C1q domain-containing protein genes

**Fig. 6:**
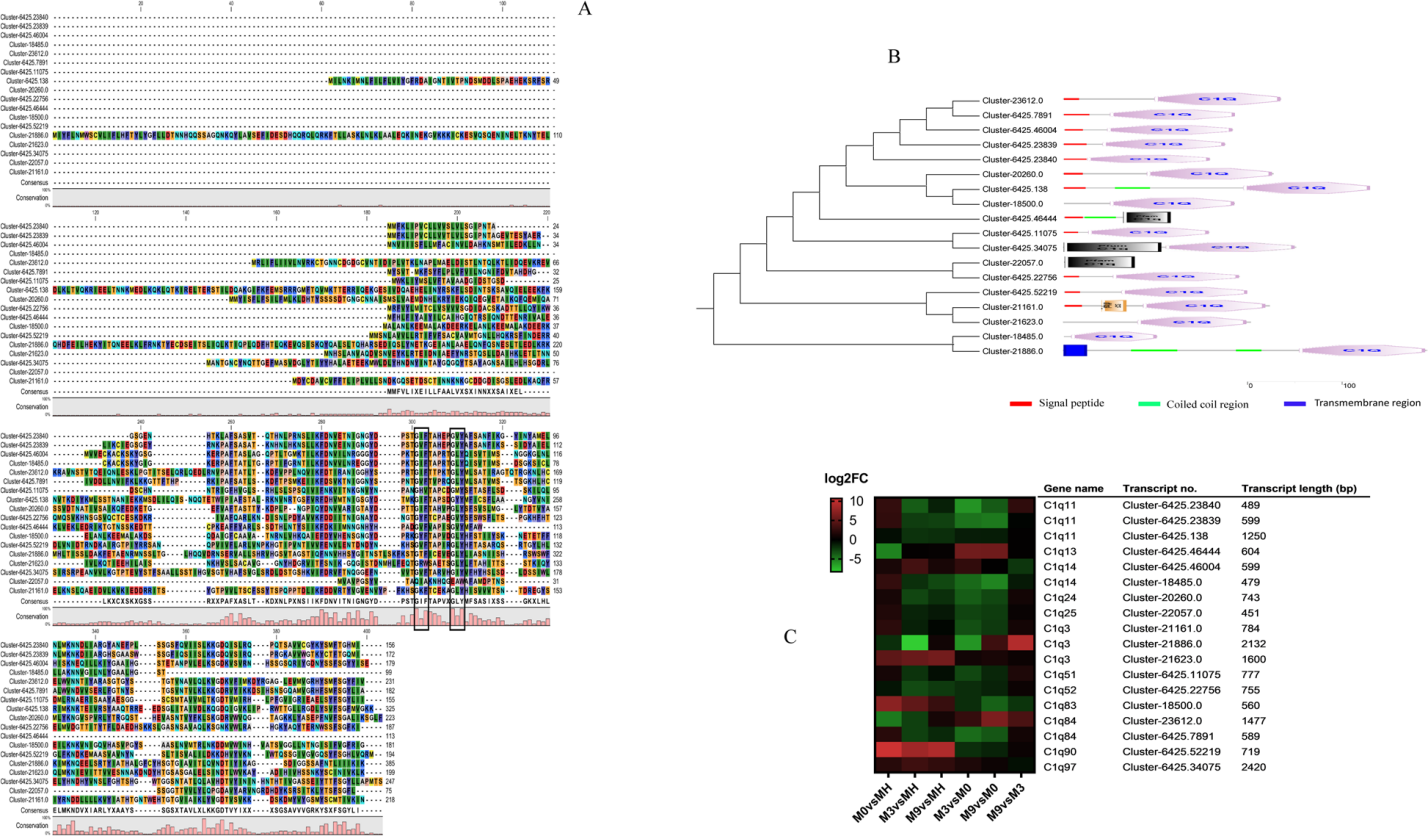
Sequence analysis and gene expression of the C1q family. **A** Protein sequence alignment of the C1q family from *G. haimaensis*. **B** Phylogenetic tree of C1q family construction and C1q protein domain analysis from *G. haimaensis*. **C** Heatmap analysis of C1q family gene expression

In the current *G. haimaensis* transcriptome, eighteen transcripts were matched to C1q domain-containing protein (C1q) sequences based on the Nr database. Based on multiple sequence alignment, those C1q proteins have a special Gly-X–Y amino acid combination that forms the collagen-like region (Fig. 6A). All the transcripts encoded the C1q domain by sequence analysis (Fig. 6B), of which only Cluster-6425.34075 encoded two C1q domains; twelve transcripts were predicted to contain an N-terminal signal peptide; three transcripts were predicted to contain coiled coil regions; and only one transcript (Cluster-21886.0) contained a transmembrane region. These C1q transcripts were affiliated with the complement system and showed differential expression patterns in the pairwise comparison group (Fig. 6C). The trend of transcript expression in M9 vs MH was similar to that in M3 vs MH. The trend of transcript expression was consistent between M3 vs M0 and M9 vs M0. The different C1q transcripts may have differential selectivity toward different environmental stresses and different abundances.

### Selection and validation of candidate genes

**Fig. 7:**
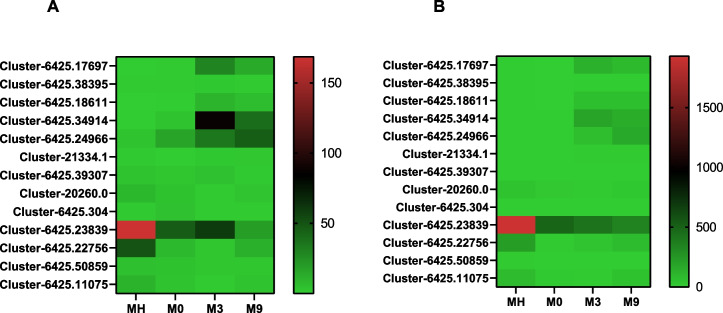
RT‒qPCR results of the thirteen candidate genes. **A** The relative expression levels using RT‒qPCR; **B** The corresponding expression levels using RNA-seq

According to the differential expression analysis, a series of genes were found to be potentially related to environmental stress. Genes involved in antioxidative defense, chaperones, the innate immune system and metabolism are considered to play vital roles in deep-sea environmental adaptation. To verify the reliability of gene expression patterns calculated with the transcriptome data, thirteen genes from this category were randomly selected for RT‒qPCR validation. The relative expression level of each gene in RT‒qPCR was similar to the expression level in transcriptome data (FPKM value) (Fig. 7). These results suggested that the gene expression profile generated from the RNA-seq data in this study was reliable.

## Discussion

The deep-sea mussel *G. haimaensis* is numerically dominant macrofauna in the Haima cold seep ecosystems. In such environments, it developed a set of adaptive mechanisms distinct from those of inshore mussels. In this study, we performed transcriptome analysis to demonstrate the molecular process response to environmental stress changes from deep-sea in situ to laboratory environments. Compared with other deep-sea mussel databases, the mean length of unigene was not only higher than the length reported, but the N50 was also significantly higher than in previously assembled transcriptomes [14, 32]. A total of 22,924 DEGs were annotated in databases, and they were mainly annotated in categories and pathways related to environmental information processing and metabolism. These results indicated that we obtained a high-quality and large transcriptome dataset of *G. haimaensis*, representing the muscle transcriptome variation from environmental stress.

Mussels may experience chilling stress changes (e.g., hydrogen sulfide concentration, water pressure, water temperature) from the deep-sea environment to the laboratory environment. To cope with environmental change, marine organisms have adapted different survival strategies. Many studies have suggested that environmental stress changes can activate oxidative stress in organisms. The cell structure was significantly damaged by oxidative stress, which could lead to protein, oxidation metabolite peroxidation, and DNA strand breaks [33–35]. According to DEG analysis, abundant genes related to antioxidants, such as glutathione s-transferase, glutathione reductase, glutathione peroxidase, thioredoxin, thioredoxin reductase, thioredoxin peroxidase, ferritin, and peroxiredoxin, were induced variably (Table S4). These genes play a key role in oxidative stress. For example, glutathione peroxidases are key enzymes that effectively protect organism cells from oxidative damage in bivalves [36, 37]. Thioredoxin can remove ROS and repair damaged proteins by oxidative stress [38]. Thioredoxin reductase is related to cellular reduction and oxidation [37, 39], while thioredoxin peroxidase, an important thiol-specific antioxidant enzyme, can protect organisms against stressful environments [40, 41]. Interestingly, most of these identified antioxidant-related genes showed negative expression regulation in M0 vs MH. Thus, the oxidative stress in the deep-sea environment may exceed that in the off-sea environment. However, oxidative stress began to increase when mussels stayed in the lab in M3 and M9. The oxidative stress was reactivated. Therefore, these genes are an important part of the tolerance mechanism to stressors in *G. haimaensis*.

In bivalves, oxidation by stress can produce DNA damage and modify DNA bases [35]. Therefore, we analyzed the mRNA expression of DNA damage- or repair-related genes (Table S5), including cell cycle checkpoint protein RAD1 (RAD1), checkpoint protein HUS1-like (HUS1), CHK1 checkpoint (CHK1), DNA damage-regulated autophagy modulator protein and DNA damage-binding protein genes. They showed different levels of expression. RAD1, HUS1 and CHK1 are critical for the cell cycle checkpoint and maintain genome integrity [42, 43]. However, RAD1, HUS1 and CHK1 exhibited opposite expression patterns. This may be due to their role diversity. The oxidative damage caused by environmental stress also induced cell apoptosis. The mRNA expression of the genes involved in apoptosis was affected, including Bcl-2, BAX, caspase 2, 3 and 8 [44, 45]. Apoptosis plays an important role in the immune response and clearing redundant or abnormal cells in organisms. We identified some genes involved in apoptosis, such as caspases, inhibitor of apoptosis protein (IAP), and the Bcl-2 family (Bcl-2 antagonist/killer protein, Bcl-2 like 2 protein and Bcl-2-associated X protein) (Table S5). IAP is involved in maintaining a balance between cell proliferation and cell death [46]. As negative regulators of apoptosis, IAPs can inhibit caspase activity [47]. In the DEG database, we found several caspase family genes, such as caspase-1, caspase-2, caspase-3/7, and caspase-8. These caspase genes have different roles in the apoptosis pathway. Caspase-2 and caspase-8 are apoptosis activators, and caspase-3/7 is an apoptosis executioner, but caspase-1 is an inflammatory mediator [48]. They directly or indirectly affect the process of apoptosis. In bivalves, the caspase family participates in development, programmed cell death, and the immune response [49]. These caspase family genes displayed differential expression patterns in MH, M0, M3, and M9. All caspases (except for Cluster-6425.20200) showed negative expression in M0 vs MH. In addition, Bcl-2 family genes can inhibit apoptosis or promote apoptosis [50]. In our analysis, the Bcl-2 gene showed positive expression in M0, M3, and M9 compared with MH. These differentially expressed genes implied that *G. haimaensis* may have to struggle to promote cellular and organismal survival by inhibiting apoptosis.

HSPs, as molecular chaperones, play a critical role in intracellular transport and protein folding [51–53]. As stressors, HSPs are induced by various stresses, such as cold or heat shock, bacterial infection, and other stresses [53]. Elevated expression of HSPs in organisms could enhance cell resistance to environmental stress [54]. In deep-sea vent/seep mussels (*B. platifrons*), the HSP70 gene appeared to be significantly expanded in the genome [55]. In addition, previous research has shown that the expression level of HSP70 positively correlates with the level of DNA strand breakage in a deep-sea mussel [56]. Therefore, these genes may provide additional genetic resources allowing deep-sea mussels to cope with abiotic stressors [55]. In our analysis, many molecular chaperone genes were apparently induced, such as HSP70, HSP90 and sacsin (Fig. 4). HSP70 is an important member of the heat shock protein family, which can repair and protect cells by rapidly regulating the cell's defense system in response to stress [57]. The HSP70 family has multiple transcripts that contain differentially conserved domains and exhibit different expression patterns, implying functional diversity. Interestingly, compared to MH, all 18 transcripts of sacsin showed negative expression in M0 but positive expression in M3 and M9. Sacsin belongs to the HSP40 family that acts as the HSP70 family of cochaperones [58–60]. Thus, the expression levels of sacsin in MH, M3, and M9 may be higher than those in M0, especially in M3. In addition, several other key proteins related to protein folding, such as XBP1, PDIA4, TXNDC5, DNAJC5B, and DNAJC5, also showed varying degrees of changes. TXNDC5 not only participates in protein folding but is also an essential antioxidant molecule [61, 62]. The results suggested that these molecular chaperone-related genes were activated and important in protecting organisms against environmental stress.

The immune system of bivalves is generally sensitive to environmental stress. Bivalves lack acquired immunity, and they must rely on innate immunity to deal with environmental stresses and pathogen invasion. In our study, we found a repertoire of immune system genes that responded to environmental change (Table S6). These genes included lysozyme, pattern recognizing proteins peptidoglycan recognition protein (PGRP), lipopolysaccharide TNF factor, bactericidal permeability increasing protein (BPI), interleukin-17 (IL-17), mytimacin, defensin, tumor necrosis factor, toll-like receptor (TLR) and C1q domain containing protein (C1qDC). Certain transcripts of lysozyme, PGRP, TLR, IL-17, mytimacin and C1qDC exhibited distinctive expression patterns in MH, M0, M3 and M9. Compared with MH, one transcript of lysozyme in M0, M3 and M9 was downregulated, and another transcript was upregulated. The lysozyme pathway was the most enriched KEGG pathway in M0 vs MH. Lysozyme is an important immune protein involved in innate immunity and possesses high antimicrobial activities in marine bivalves. Toll-like receptors are a large family of pattern recognition receptors that recognize pathogens in mussels [63]. We found that most Toll-like receptor transcripts showed negative expression in M0, M3, and M9 compared with MH. This may be related to the diverse pathogenic bacteria in cold springs. To defend against infection by pathogenic microorganisms, Toll-like receptors always maintain high expression levels in MHs. A group of C1qDC proteins that contains Gly-X–Y motifs shows the conserved C1Q domain. This unique structure can increase its thermal stability [64]. In *Crassostrea gigas*, C1qDCs not only recognize pathogens but also enhance phagocytosis of phagocytic cells by interacting with receptors on the cell surface [65, 66]. Surprisingly, most C1qDC transcripts appeared to have a high expression level in M0. That is, the complement system played an essential role in M0.

To cope with stress, marine organisms have adapted different metabolic strategies [67–69]. This study altered genes and pathways involved in lipid metabolism and glycolysis (Table S7). Lipid metabolism was the obviously enriched KEGG pathway in all pairwise comparison groups. Lipids are an essential component of membranes. By regulating phospholipid membrane proteins, deep-sea organisms can alter membrane fluidity in response to environmental stress [70]. In our study, the expression of lipid metabolism genes began to decrease from MH to M0 and then increased again from M0 to M3 or M9 (Fig. 5). This indicated that *G. haimaensis* tried to maintain cell membrane stability to cope with environmental stress. Glycolysis is the main pathway of energy generation in both vertebrates and invertebrates. Hypoxia stress is a feature of seep ecosystems and is connected with the activation of glycolysis [71–73]. Under air exposure stress, glycolysis-related genes in *C. gigas* have been found to increase significantly, including phosphofructokinase, glyceraldehyde-3-phosphate dehydrogenase, and hexokinase [74]. In our study, the key enzymes of glycolysis, such as pyruvate kinase and hexokinase, were significantly upregulated in M0, M3, and M9 compared to MH, suggesting that glycolysis was activated under stress, and *G. haimaensis* had to increase its energy supply to resist environmental stress from the deep-sea in situ environment to the laboratory environment.

## Conclusion

This study investigated the gene expression patterns from the deep sea in situ to the laboratory for the cold seep mussel *G. haimaensis* using transcriptomic approaches. This result showed that *G. haimaensis* may have to change its gene expression pattern to respond to the environmental stress change from the deep sea to the laboratory. These DEGs were involved in antioxidative defense, apoptosis, chaperones, the innate immune system and metabolism. Environmental information processing and metabolism pathways may play an important role in the response to environmental stress because of the significant enrichment of KEGG pathways in all pairwise comparison groups. Overall, our transcriptomic data will serve as an essential reference for future research on the adaptation of the mussel *G. haimaensis* from cold seep environments to laboratory environments.

## Supplementary Information

**Additional file 1: Fig. S1.** Blast of NR database. **Fig. S2.** GO annotation classification. **Fig. S3.** KEGG pathway classification statistics. A: Cellular Processes; B: Environmental Information Processing; C: Genetic Information Processing; D: Metabolism; E: Organismal Systems. **Fig. S4.** KOG annotation classification. **Fig. S5.** Go annotation of DEGs. A: The enrichment of down and up gene in M0 vs MH; B: The enrichment of down and up gene in M3 vs MH; C: The enrichment of down and up gene in M9 vs MH; D: The enrichment of down and up gene in M3vs M0; E: The enrichment of down and up gene in M9 vs M0; F: The enrichment of down and up gene in M9 vs M3.

**Additional file 2: Table S1.** Nucleotide sequences of the primers for qPCR. **Table S2.** Summary of sequencing data. **Table S3.** Annotation of databases. **Table S4.** Oxidative stress response gene. **Table S5.** DNA repair and apoptosis genes. **Table S6.** Immune system genes. **Table S7.** Metabolism genes.

## Data Availability

The raw sequence data reported in this paper have been deposited in the Science Data Bank (https://www.scidb.cn/anonymous/Wk5Cbm1t).
