# Supplementary material for: Transcriptome analysis of gene expression profiling from the deep sea in situ to the laboratory for the cold seep mussel Gigantidas haimaensis
Source: BMC Genomics. 2022 Dec 14;23:828. doi: 10.1186/s12864-022-09064-9 (PMC9749274; doi:10.1186/s12864-022-09064-9)

**Fig. S1 Blast of NR database**


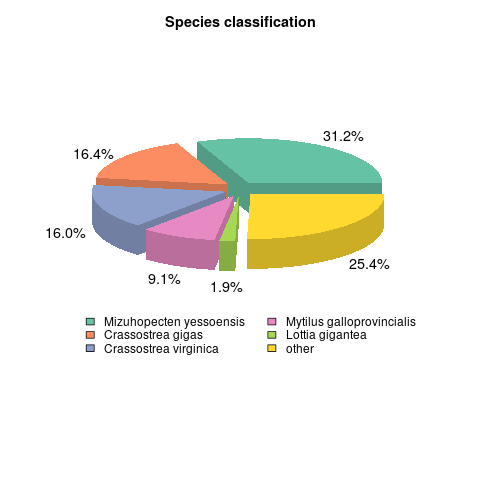


**Fig. S2 GO annotation classification**


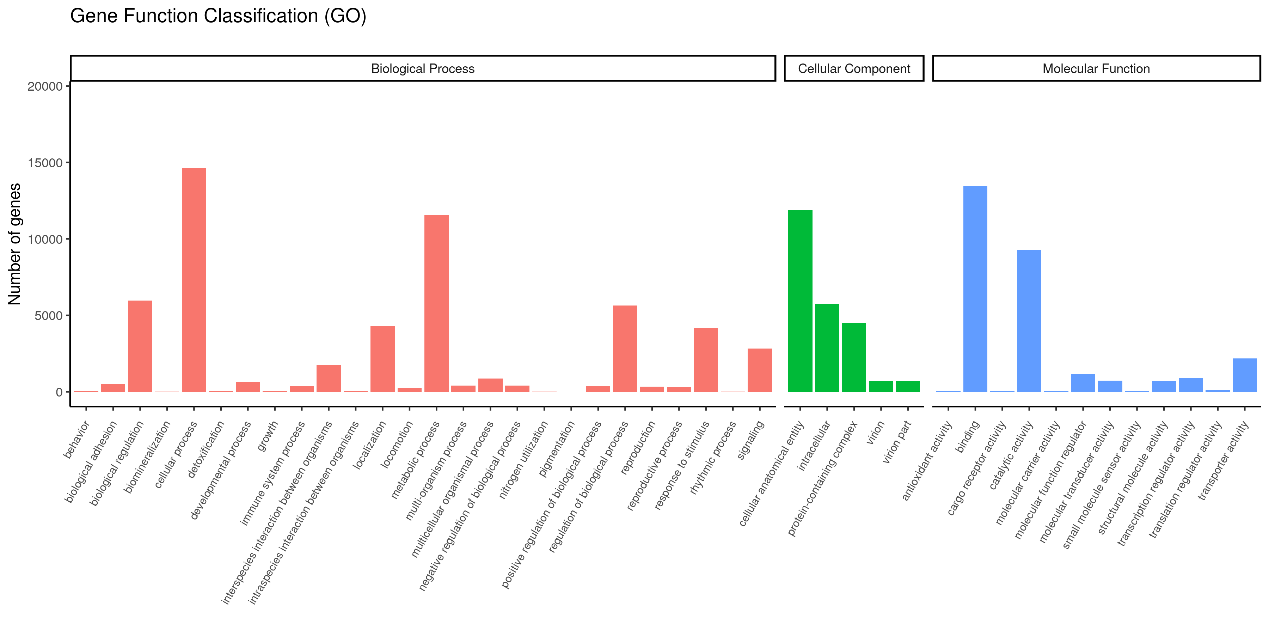


**Fig. S3 KEGG pathway classification statistics. A: Cellular Processes; B: Environmental Information Processing; C: Genetic Information Processing; D: Metabolism; E: Organismal Systems.**


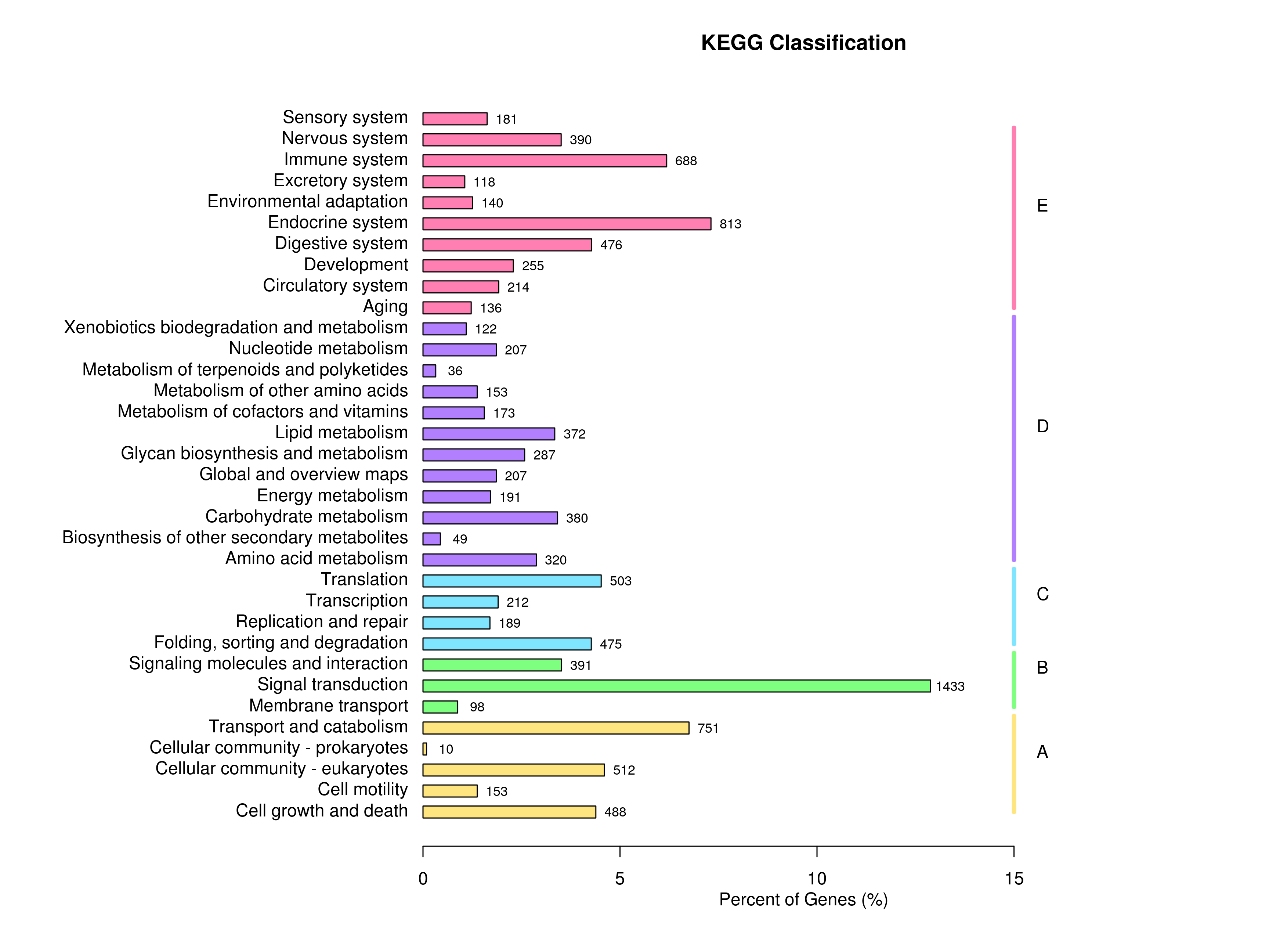


**Fig. S4 KOG annotation classification**


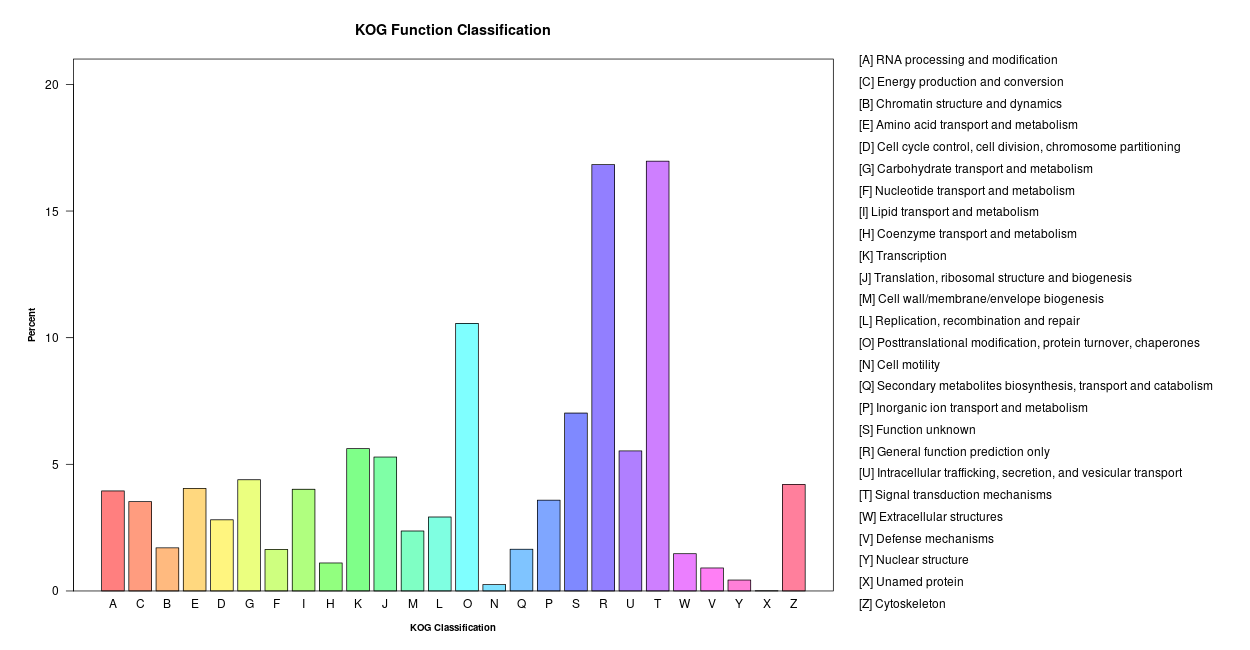


**Fig. S5 Go annotation of DEGs. A: The enrichment of down and up gene in M0 vs MH; B: The enrichment of down and up gene in M3 vs MH; C: The enrichment of down and up gene in M9 vs MH; D: The enrichment of down and up gene in M3vs M0; E: The enrichment of down and up gene in M9 vs M0; F: The enrichment of down and up gene in M9 vs M3.**

A


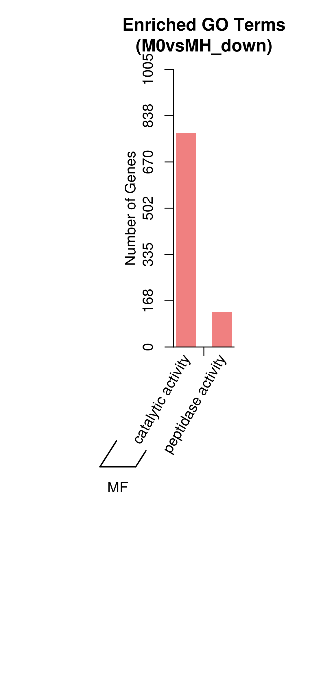

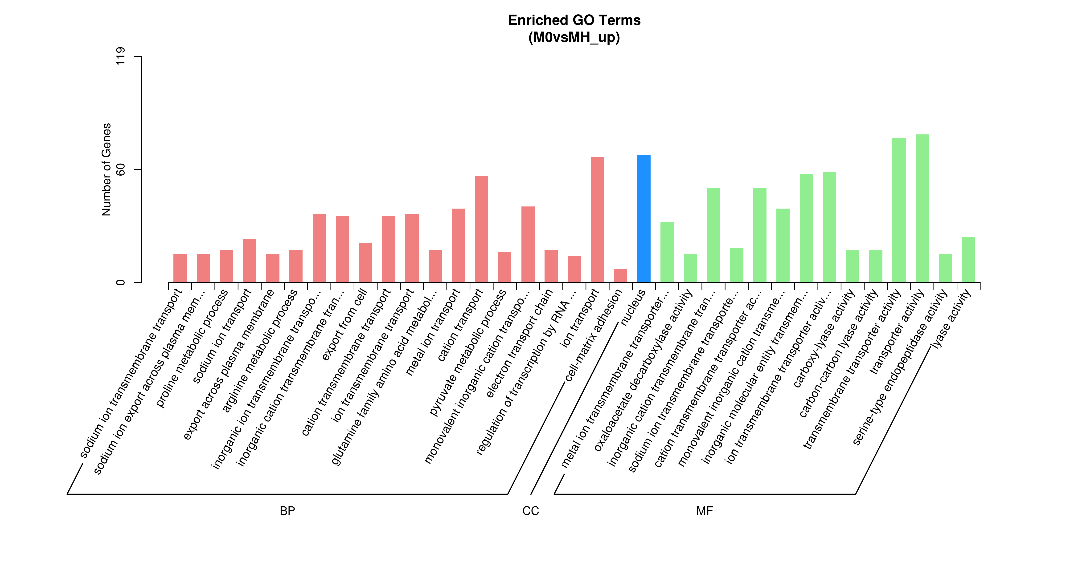


B


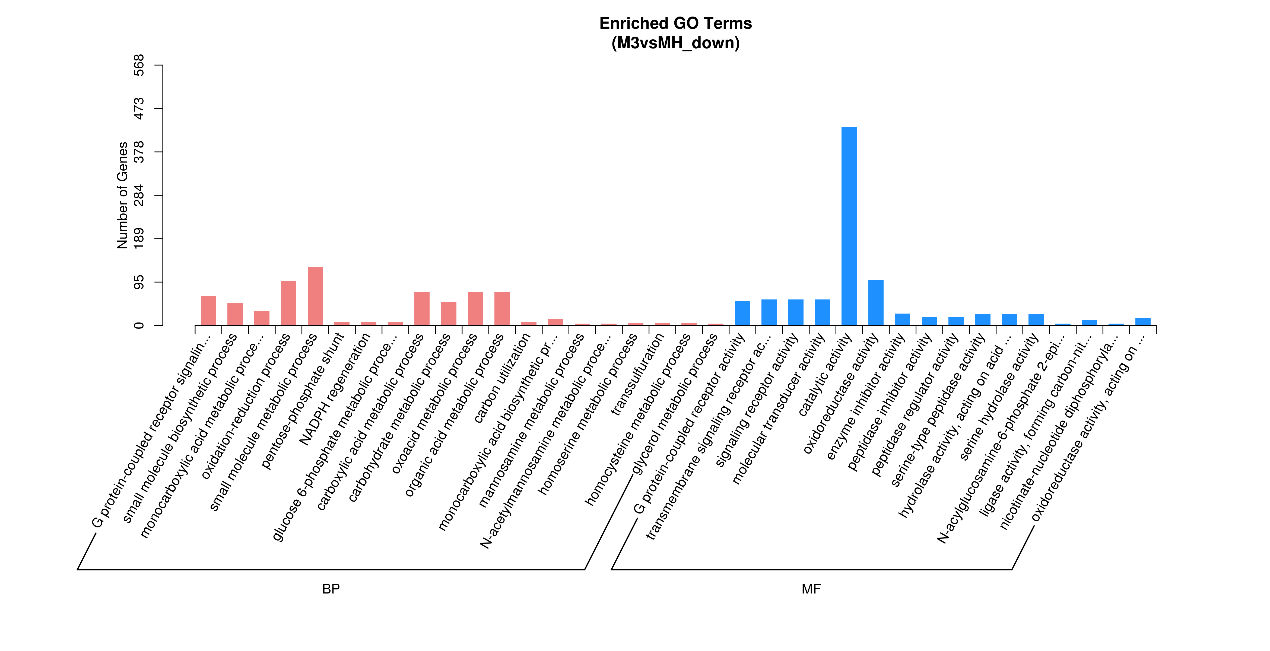


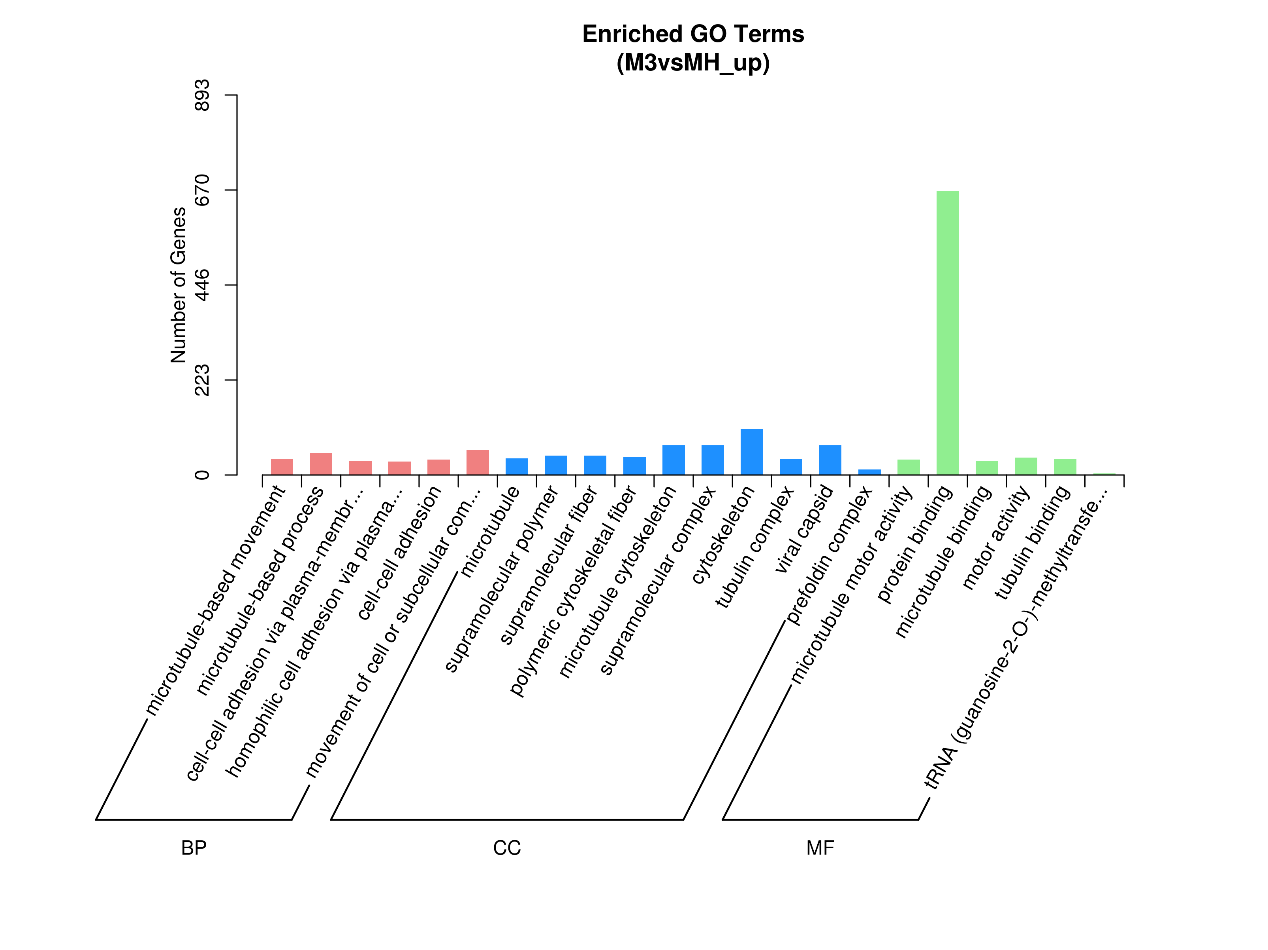


C


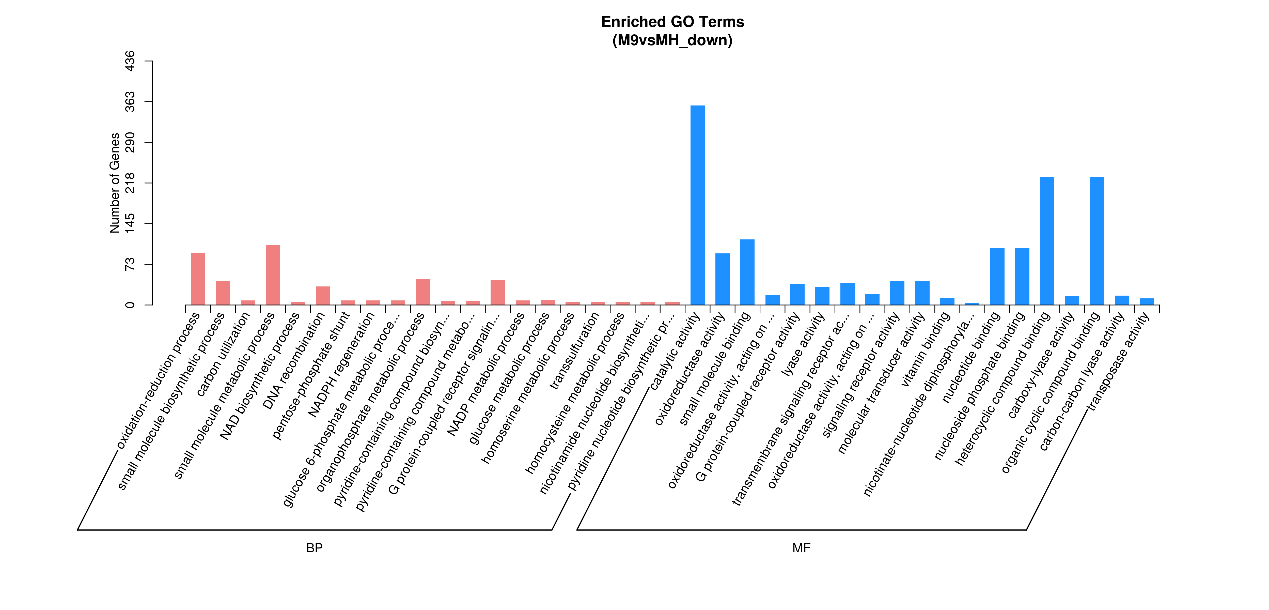


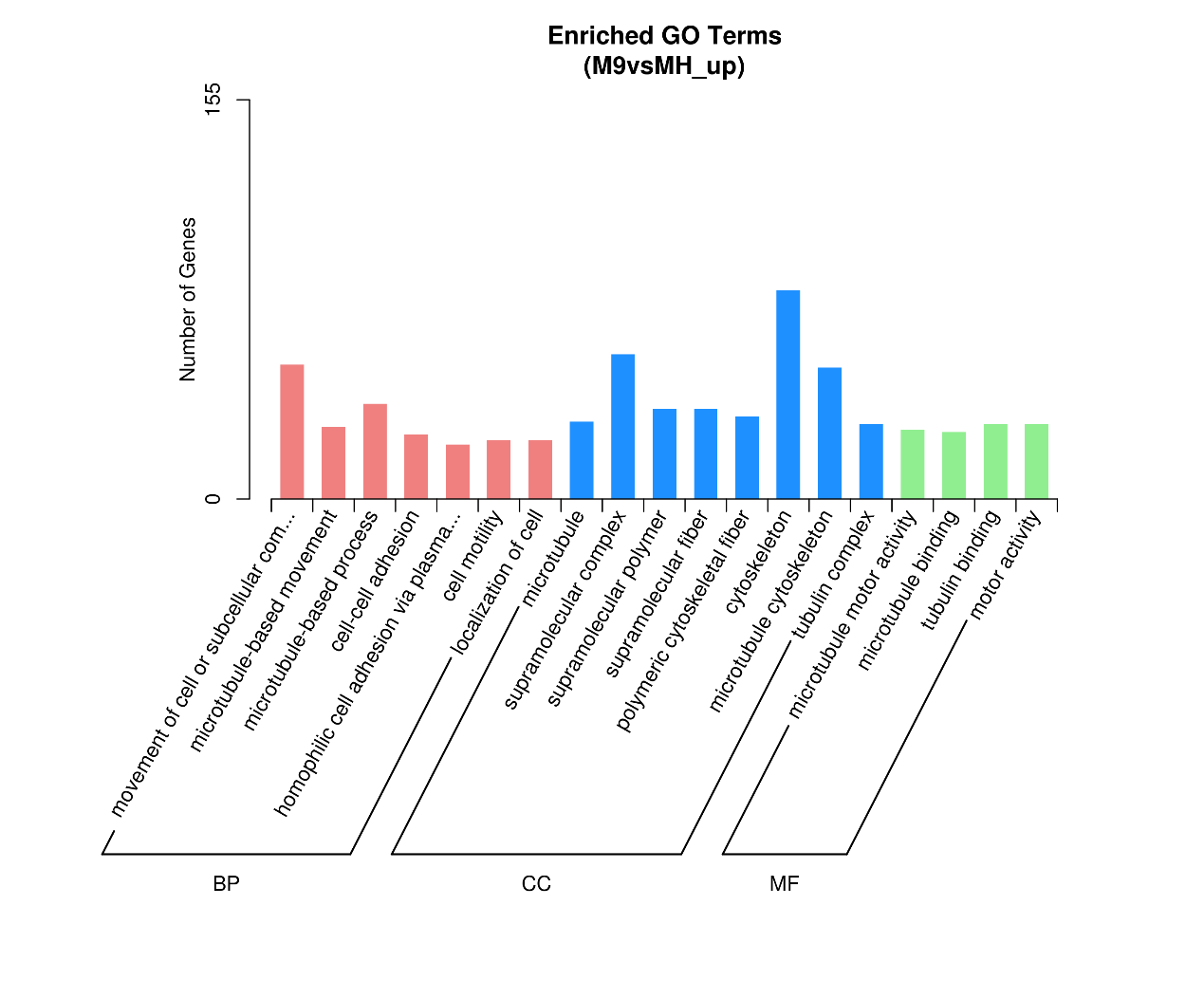


D


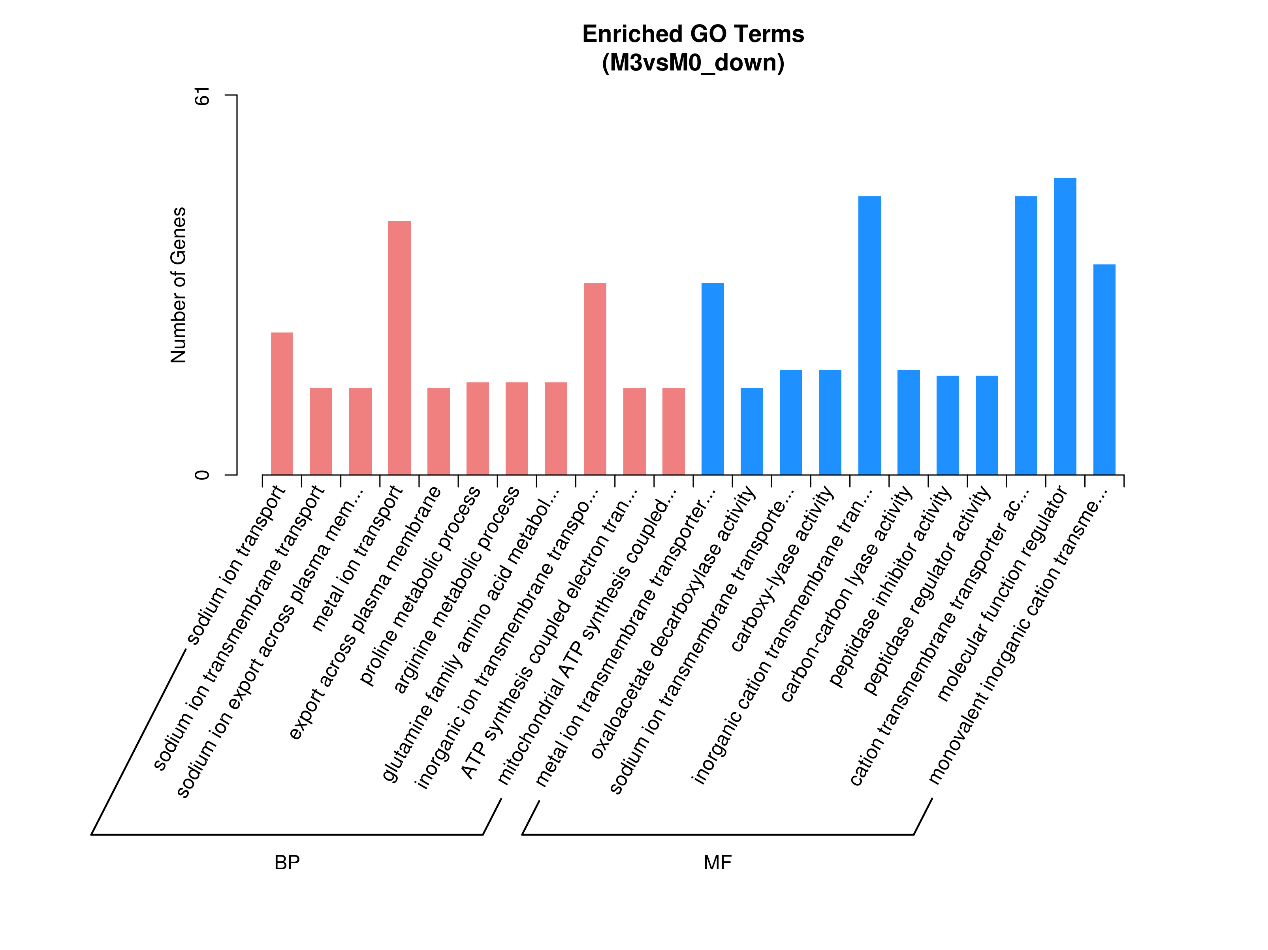


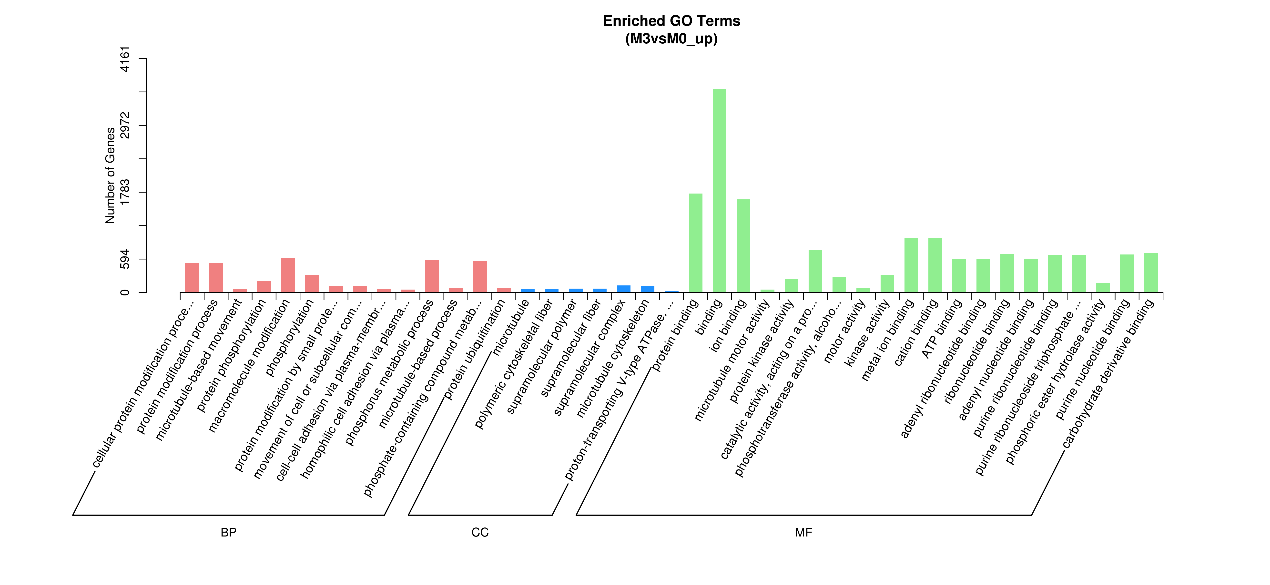


E


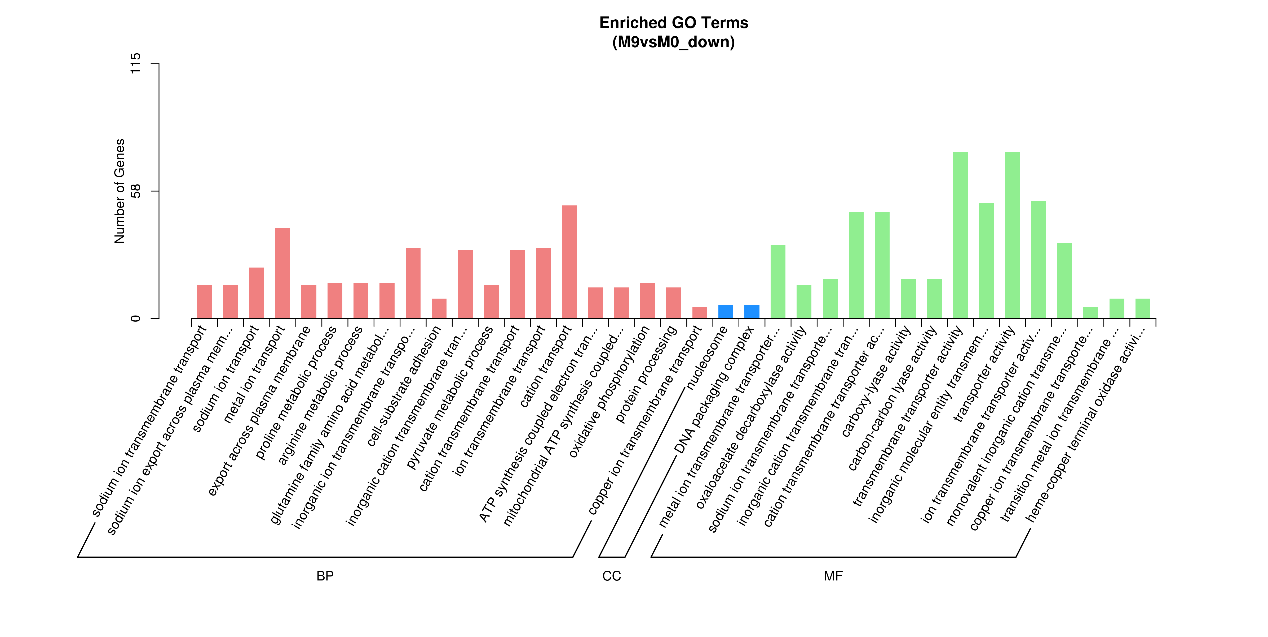


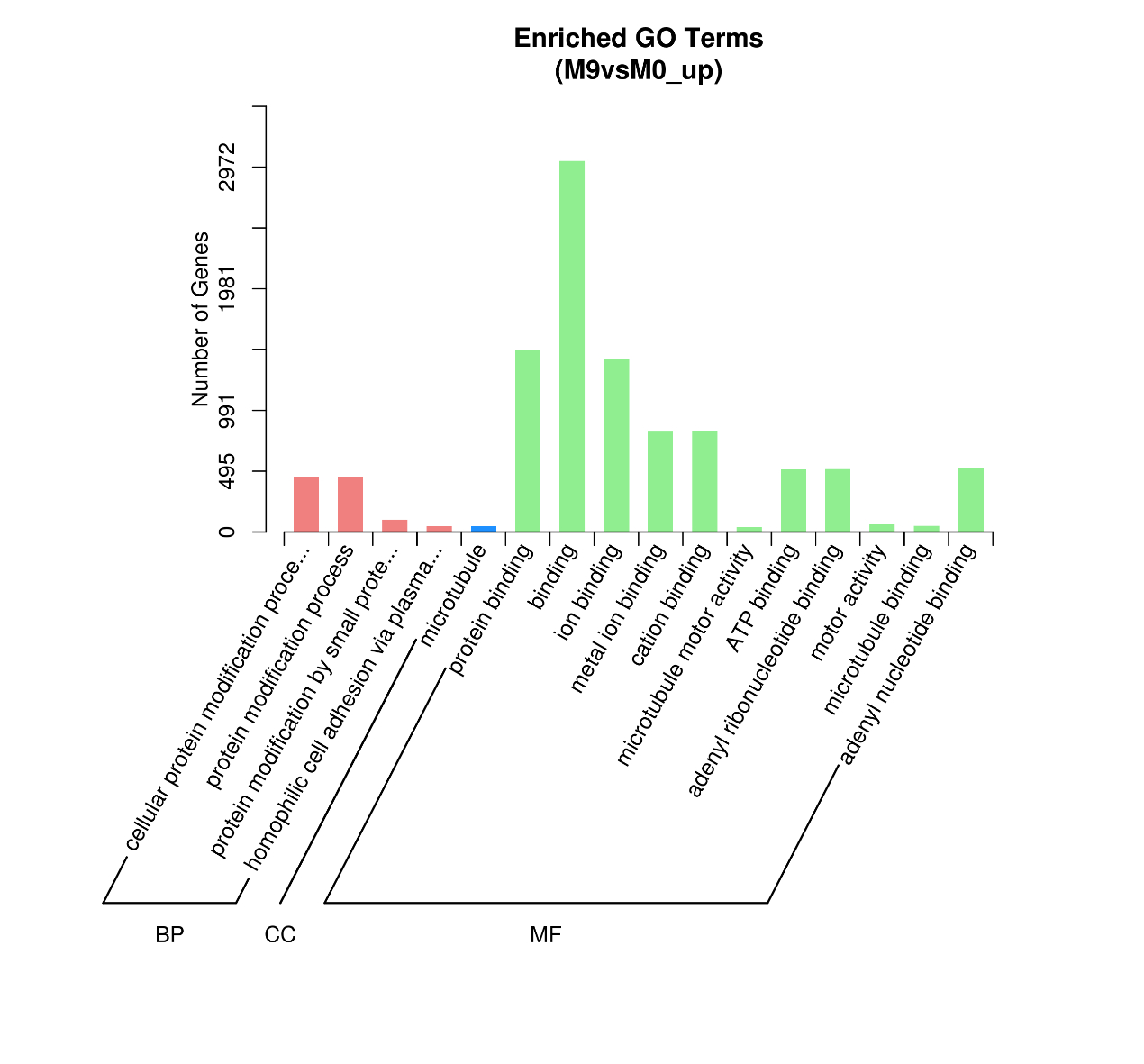


F


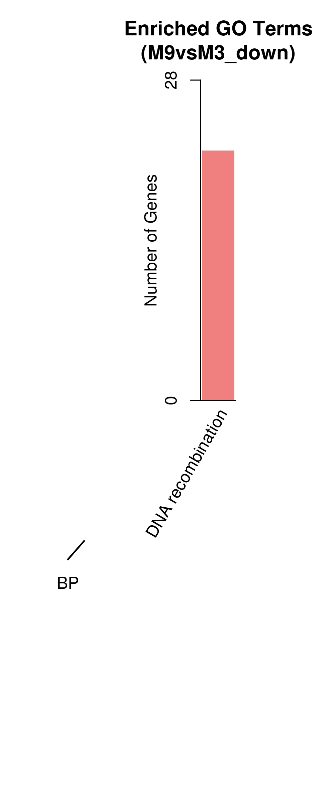


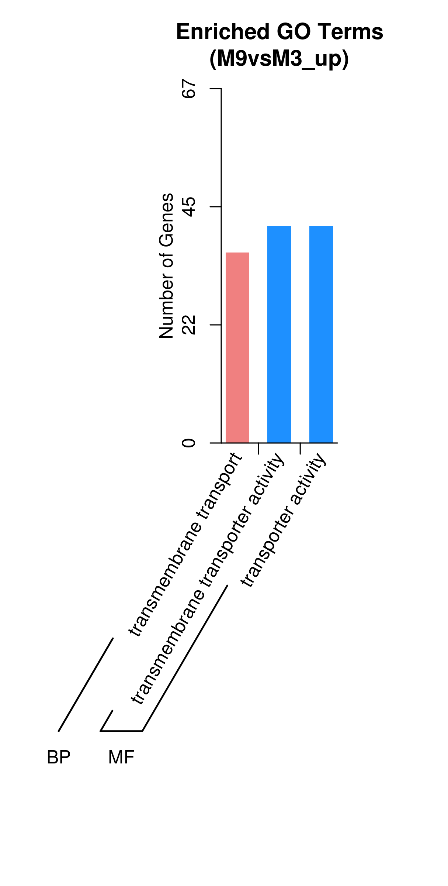

Supplement: Supplementary file 1 — Additional file 1: Fig. S1. Blast of NR database. Fig. S2. GO annotation classification. Fig. S3. KEGG pathway classification statistics. A: Cellular Processes; B: Environmental Information Processing; C: Genetic Information Processing; D: Metabolism; E: Organismal Systems. Fig. S4. KOG annotation classification. Fig. S5. Go annotation of DEGs. A: The enrichment of down and up gene in M0 vs MH; B: The enrichment of down and up gene in M3 vs MH; C: The enrichment of down and up gene in M9 vs MH; D: The enrichment of down and up gene in M3vs M0; E: The enrichment of down and up gene in M9 vs M0; F: The enrichment of down and up gene in M9 vs M3. [file 12864_2022_9064_MOESM1_ESM.docx]
