# Supplementary material for: Transcriptome analysis of gene expression profiling from the deep sea in situ to the laboratory for the cold seep mussel Gigantidas haimaensis
Source: BMC Genomics. 2022 Dec 14;23:828. doi: 10.1186/s12864-022-09064-9 (PMC9749274; doi:10.1186/s12864-022-09064-9)
Supplement: Supplementary file 2 — Additional file 2: Table S1. Nucleotide sequences of the primers for qPCR. Table S2. Summary of sequencing data. Table S3. Annotation of databases. Table S4. Oxidative stress response gene. Table S5. DNA repair and apoptosis genes. Table S6. Immune system genes. Table S7. Metabolism genes. [file 12864_2022_9064_MOESM2_ESM.docx]

**Supporting information**

**Table S1 Nucleotide sequences of the primers for qPCR**

| Selected genes | Primers sequences(5’-3’) |
| --- | --- |
| Cluster-6425.138-F119 | ATCGTATGCTGCCCAAACCA |
| Cluster-6425.138-R119 | ACAATCCCGTAGTCCATCGG |
| Cluster-6425.11075-F143 | AGCTCCGTGTGATGGAATGT |
| Cluster-6425.11075-R143 | CAACAGCAGTCATGGAACACG |
| Cluster-6425.22756-F168 | CACCAGGCAAGCATTTCCAC |
| Cluster-6425.22756-R168 | CGTACTTTCCATGAGCCCGT |
| Cluster-6425.23839-F113 | AATACGCCTGTGCTAGGGTC |
| Cluster-6425.23839-R113 | GCATTCTCTGCATCGGCAAC |
| Cluster-20260.0-F98 | TGGCCTTCACAGCCTCTACT |
| Cluster-20260.0-R98 | AGGATCGTATGCCGTTCCAA |
| Cluster-6425.11424-F0 | GGTGCCCCACGATTACCAAT |
| Cluster-6425.11424-R0 | TGAAGTGGTCCCTGCTTGAC |
| Cluster-6425.50859-F94 | TTAACACCCGAAGGCACTCC |
| Cluster-6425.50859-R94 | GTGGGTGTGTTTTTCGCCTC |
| Cluster-6425.304-F147 | CCCCACTTCTACACTAGGCA |
| Cluster-6425.304-R147 | GGACGTACAAAGACGGTGGA |
| Cluster-6425.39307-F162 | GGCAGCTAATGTTGGTGCTG |
| Cluster-6425.39307-R162 | GGCCCAAGCGTGTCATACAA |
| Cluster-21334.1-F161 | CAACTGCTGGAGACACCCAT |
| Cluster-21334.1-R161 | CAATGTCCGTTTTGCCCGTT |
| Cluster-6425.24966-F138 | TTCGTGGACAATCAGGCAGG |
| Cluster-6425.24966-R138 | TACCACGATTTGAGGCACCC |
| Cluster-6425.34914-F131 | TTGATGTCGCTCCGTTGTCA |
| Cluster-6425.34914-R131 | TGCCGGCTGATTGTCTGAAT |
| Cluster-6425.17697-F164 | TGACCGAGGACGACTGAGTA |
| Cluster-6425.17697-R164 | ATTTGTCGCCAGAGTCACCA |
| Cluster-6425.18611-F104 | CAAGCCTCTCAGTGTCGGTAA |
| Cluster-6425.18611-R104 | CTGTGTCGGAGTTTTCCAGCA |
| Cluster-6425.38395-F154 | ACGTGGTAATGCTACCAAGCC |
| Cluster-6425.38395-R154 | GTGGGCTTCGTGGTTCGTTA |
| Cluster-6425.27802-b-actinF141 | ACGTTTTACCTGGGCTTCGT |
| Cluster-6425.27802-b-actinR141 | AAATCAGGTTCGGCTGGTGA |

**Table S2 Summary of sequencing data**

| sample | raw_reads | clean_reads | clean_bases | error_rate | Q20 | Q30 | GC_pct |
| --- | --- | --- | --- | --- | --- | --- | --- |
| MH_1 | 21628147 | 21246721 | 6.37G | 0.03 | 97.95 | 93.91 | 38.51 |
| MH_2 | 21503006 | 21204027 | 6.36G | 0.03 | 97.85 | 93.57 | 38.1 |
| M0_1 | 21670255 | 21219852 | 6.37G | 0.03 | 97.29 | 92.6 | 33.51 |
| M0_2 | 22708182 | 22196737 | 6.66G | 0.03 | 97.15 | 92.48 | 33.29 |
| M0_3 | 23517360 | 22930484 | 6.88G | 0.03 | 97.25 | 92.62 | 33.47 |
| M3_1 | 21534561 | 21044784 | 6.31G | 0.03 | 97.76 | 93.46 | 36.58 |
| M3_2 | 22532966 | 22016286 | 6.6G | 0.03 | 97.78 | 93.59 | 36.1 |
| M3_3 | 21474057 | 20934918 | 6.28G | 0.03 | 98.01 | 93.95 | 37.08 |
| M9_1 | 22675741 | 22049256 | 6.61G | 0.03 | 97.71 | 93.43 | 36.36 |
| M9_2 | 22812576 | 22204440 | 6.66G | 0.03 | 97.83 | 93.63 | 37.26 |
| M9_3 | 22096079 | 21567334 | 6.47G | 0.03 | 97.73 | 93.37 | 36.66 |

**Table S3 Annotation of databases**

|  | Number of Unigenes | Percentage (%) |
| --- | --- | --- |
| Annotated in NR | 27733 | 32.99 |
| Annotated in NT | 14541 | 17.3 |
| Annotated in KO | 10332 | 12.29 |
| Annotated in SwissProt | 17823 | 21.2 |
| Annotated in PFAM | 25287 | 30.08 |
| Annotated in GO | 25283 | 30.08 |
| Annotated in KOG | 9041 | 10.75 |
| Annotated in all Databases | 2687 | 3.19 |
| Annotated in at least one Database | 42169 | 50.17 |
| Total Unigenes | 84050 | 100 |

**Table S4 Oxidative stress response gene**

| **Transcript no.** | **Transcript length (bp)** | **log2FC M0vsMH** | **log2FC M3vsMH** | **log2FC M9vsMH** | **log2FC M3vsM0** | **log2FC M9vsM0** | **log2FC M9vsM3** | **Nr annotation** |
| --- | --- | --- | --- | --- | --- | --- | --- | --- |
| **Thioredoxin/Thioredoxin reductase/thioredoxin peroxidase** | | | | | | | | |
| Cluster-6425.1487 | 1988 | -2.5557 | 0.48436 | -0.0011136 | 3.3331↑ | 2.7117 | -0.48882 | thioredoxin reductase 2, mitochondrial-like isoform X2 [Mizuhopecten yessoensis] |
| Cluster-6425.13651 | 2137 | -1.7636 | 0.79709 | 0.82637 | 2.8558↑ | 2.743↑ | 0.051912 | thioredoxin reductase 1, cytoplasmic-like [Mizuhopecten yessoensis] |
| Cluster-6425.24215 | 1222 | -1.9463 | 0.39717 | 0.39903 | 2.6579↑ | 2.5008↑ | 0.050683 | PREDICTED: thioredoxin-like protein 1 [Crassostrea gigas] |
| Cluster-6425.28811 | 1586 | -2.1941 | -0.37745 | -0.27662 | 2.1758↑ | 2.1336↑ | 0.12229 | thioredoxin peroxidase [Cristaria plicata] |
| **Peroxiredoxin** | | | | | | | | |
| Cluster-6425.30578 | 1961 | -2.1326 | 0.57502 | 0.26557 | 3.0585↑ | 2.5974↑ | -0.26462 | peroxiredoxin-2-like [Mizuhopecten yessoensis] |
| Cluster-6425.34733 | 1002 | -3.0734 | -0.37889 | -0.23994 | 3.041↑ | 3.0452↑ | 0.16312 | peroxiredoxin-5, mitochondrial-like [Crassostrea virginica] |
| **Ferritin** | | | | | | | | |
| Cluster-14200.0 | 935 | -1.4756 | 3.6476↑ | 1.2759 | 5.3189↑ | 2.8441 | -2.3371 | ferritin [Rhipicephalus microplus] |
| Cluster-6425.1044 | 1195 | -6.1428↓ | -9.7957↓ | -9.7602↓ | -3.2316 | -3.3338 | NA | ferritin peptide [Penaeus indicus] |
| Cluster-6425.3311 | 1110 | -3.0343 | -0.38854 | 0.12449 | 2.9481↑ | 3.2997↑ | 0.53331 | soma ferritin-like [Crassostrea gigas] |
| Cluster-6425.4291 | 1167 | 4.1311↑ | -3.4455↓ | 1.274 | -7.2807 | -2.7482 | 4.7483↑ | soma ferritin [Crassostrea gigas] |
| **Glutathione S-transferase/glutathione reductase/glutathione peroxidase** | | | | | | | | |
| Cluster-18078.0 | 890 | 1.2365 | -1.3522 | 3.4484 | -2.2699 | 2.3328 | 4.8294↑ | glutathione S-transferase sigma 3 [Mytilus galloprovincialis] |
| Cluster-6425.878 | 1126 | -0.8409 | -2.1985↓ | -0.8488 | -1.0097 | 0.18201 | 1.377 | glutathione S-transferase sigma 3 [Mytilus galloprovincialis] |
| Cluster-6425.18326 | 1040 | -2.5999↓ | -0.74804 | -1.2892 | 2.1745↑ | 1.4879 | -0.55732 | PREDICTED: microsomal glutathione S-transferase 3 [Crassostrea gigas] |
| Cluster-6425.16896 | 1028 | -2.3883 | -0.29849 | -0.19959 | 2.4101↑ | 2.3556↑ | 0.089988 | glutathione S-transferase-like [Pomacea canaliculata] |
| Cluster-6425.26503 | 2612 | -1.5609 | 0.50626 | 0.15338 | 2.4182↑ | 1.9676 | -0.36801 | glutathione S-transferase Mu 3-like [Crassostrea virginica] |
| Cluster-24479.0 | 511 | 6.1574↑ | 0.24445 | 0.27197 | -5.6164↓ | -5.7282↓ | 0.044664 | glutathione S-transferase sigma 1 [Mytilus galloprovincialis] |
| Cluster-6425.22482 | 763 | 1.3629 | -3.4135 | 0.40764 | -4.4603↓ | -0.67672 | 3.9351↑ | microsomal glutathione S-transferase 3 isoform c [Daphnia magna] |
| Cluster-6425.22480 | 326 | 0.98991 | -5.5198 | 0.28055 | -6.1795↓ | -0.47459 | 5.8554↑ | microsomal glutathione S-transferase 3-like [Folsomia candida] |
| Cluster-25405.0 | 890 | 10.175↑ | 2.3307 | 1.0949 | -7.5255 | -8.9034 | -1.2623 | PREDICTED: glutathione peroxidase-like [Crassostrea gigas] |
| Cluster-6425.8592 | 1217 | 2.345 | 3.8494↑ | 3.7253 | 1.8441 | 1.5559 | -0.050274 | glutathione peroxidase-like [Pomacea canaliculata] |
| Cluster-6425.37111 | 2200 | -1.1451 | 0.69217 | 0.45393 | 2.1596↑ | 1.7863 | -0.22956 | glutathione reductase [Haliotis discus discus] |

**Table S5 DNA repair and apoptosis genes**

| **Transcript no.** | **Transcript length (bp)** | **log2FC M0vsMH** | **log2FC M3vsMH** | **log2FC M9vsMH** | **log2FC M3vsM0** | **log2FC M9vsM0** | **log2FC M9vsM3** | **Nr annotation** |
| --- | --- | --- | --- | --- | --- | --- | --- | --- |
| **Checkpoint protein** | | | | | | | | |
| Cluster-23825.0 | 1045 | -1.6882 | 0.4779 | 0.48994 | 2.5083↑ | 2.3501↑ | 0.051823 | Cell cycle checkpoint protein RAD1 [Mizuhopecten yessoensis] |
| Cluster-6425.10050 | 1186 | -5.3053↓ | 1.8365 | 1.4641 | 7.4618 | 6.9077↑ | -0.31166 | PREDICTED: checkpoint protein HUS1-like [Crassostrea gigas] |
| Cluster-6425.30471 | 383 | 2.0393 | -2.1776↓ | -1.1243 | -3.8737↓ | -3.0105↓ | 1.0681 | CHK1 checkpoint [Echinococcus multilocularis] |
| **DNA damage-regulated autophagy modulator protein/DNA damage-binding protein** | | | | | | | | |
| Cluster-8154.1 | 858 | 5.662 | 3.7889 | 5.1936 | -1.5492 | -0.29089 | 1.3755 | DNA damage-regulated autophagy modulator protein 2 [Mizuhopecten yessoensis] |
| Cluster-6425.12520 | 4078 | -1.3474 | 0.32416 | 0.27891 | 2.0059↑ | 1.8191 | -0.020924 | DNA damage-binding protein 1-like isoform X2 [Mizuhopecten yessoensis] |
| **Caspase** | | | | | | | | |
| Cluster-6425.46812 | 585 | -2.3174 | 2.1284 | 3.1611↑ | 4.7676↑ | 5.7443↑ | 1.0302 | caspase-2, partial [Mytilus galloprovincialis] |
| Cluster-6425.20200 | 1117 | 2.455 | 2.2099↑ | 0.37624 | 0.080333 | -1.8902 | -1.8445 | Caspase-2 [Mizuhopecten yessoensis] |
| Cluster-6425.16158 | 682 | -2.1031 | -0.3993 | -0.49837 | 2.0557↑ | 1.8197 | -0.088804 | caspase-2, partial [Mytilus galloprovincialis] |
| Cluster-6425.22525 | 836 | -3.1815 | 1.8574 | 0.81456 | 5.2987↑ | 4.1374↑ | -1.0412 | caspase 2 [Mytilus galloprovincialis] |
| Cluster-6425.17103 | 938 | -2.4435↓ | -0.76608 | -0.49461 | 2.018↑ | 2.158↑ | 0.2885 | caspase-2, partial [Mytilus galloprovincialis] |
| Cluster-6425.21219 | 377 | -3.2797 | 1.4365 | 1.9417 | 5.0239↑ | 5.379↑ | 0.48534 | caspase 3/7-3 [Mytilus galloprovincialis] |
| Cluster-6425.15339 | 733 | -1.2361 | 1.3461 | 0.73133 | 2.8938↑ | 2.1363 | -0.59378 | caspase 3/7-2 [Mytilus galloprovincialis] |
| Cluster-6425.18241 | 1778 | -2.2842↓ | 0.40288 | -0.16873 | 3.0189↑ | 2.329↑ | -0.57027 | caspase-1-like isoform X3 [Mizuhopecten yessoensis] |
| Cluster-6425.14149 | 1694 | -1.7551 | 0.20676 | -0.020497 | 2.2747↑ | 1.9109 | -0.22378 | caspase-1 [Crassostrea gigas] |
| Cluster-24295.0 | 1243 | -1.768 | -3.5972↓ | -1.8961 | -1.4758 | 0.090609 | 1.7311 | caspase-8 [Tubifex tubifex] |
| Cluster-19225.2 | 1457 | -0.28408 | -1.7558 | -2.8342↓ | -1.1465 | -2.2542 | -1.097 | caspase-8-like protein [Mytilus californianus] |
| Cluster-6425.20476 | 3749 | -1.7788 | 0.065357 | -0.3487 | 2.1865↑ | 1.6446 | -0.41145 | caspase-8-like protein [Mytilus californianus] |
| Cluster-24576.0 | 2219 | -1.8298 | -0.093073 | -0.41097 | 2.0601↑ | 1.6022 | -0.29289 | caspase-8 [Mytilus californianus] |
| **Inhibitor of apoptosis** | | | | | | | | |
| Cluster-6425.40263 | 3144 | -1.5461 | 1.2108 | 0.91813 | 3.1139↑ | 2.6951↑ | -0.26067 | apoptosis inhibitor 5-like [Mizuhopecten yessoensis] |
| Cluster-6425.15679 | 303 | NA | 6.0594↑ | 5.3048↑ | 5.1274↑ | 4.2505↑ | -0.76047 | inhibitor of apoptosis, partial [Mytilus galloprovincialis] |
| Cluster-6425.15220 | 1247 | -0.073831 | 5.0084↑ | 3.2991 | 5.4707↑ | 3.6631 | -1.6898 | inhibitor of apoptosis, partial [Mytilus galloprovincialis] |
| **Bcl-2 protein/ Bcl-2 antagonist/killer protein/ Bcl-2-associated X protein** | | | | | | | | |
| Cluster-6425.47889 | 1586 | 1.4866 | 3.5701↑ | 3.1601↑ | 2.4065↑ | 1.8359 | -0.34612 | Bcl-2 like 2 protein [Perna viridis] |
| Cluster-6425.606 | 2013 | -3.8299 | 0.85973 | -0.17419 | 5.0226↑ | 3.8847↑ | -0.98932 | Bcl-2 antagonist/killer protein [Perna viridis] |
| Cluster-6425.40972 | 1908 | -1.9755 | 0.46357 | 0.27897 | 2.7866↑ | 2.4793↑ | -0.16205 | Bcl-2-associated X protein [Mytilus galloprovincialis] |

**Table S6 Immune system genes**

| **Transcript no.** | **Transcript length (bp)** | **log2FC M0vsMH** | **log2FC M3vsMH** | **log2FC M9vsMH** | **log2FC M3vsM0** | **log2FC M9vsM0** | **log2FC M9vsM3** | **Nr annotation** |
| --- | --- | --- | --- | --- | --- | --- | --- | --- |
| **Lysozyme** | | | | | | | | |
| Cluster-6425.4742 | 1527 | -5.0434↓ | -3.0378↓ | -1.6556 | 2.3473 | 3.6811 | 1.4118 | lysozyme [*Bathymodiolus thermophilus*] |
| Cluster-6425.37109 | 843 | NA | 5.9661↑ | 1.2843 | 5.0648 | 0.14567 | -4.7447↓ | lysozyme, partial [*Mytilus* *galloprovincialis*] |
| **Peptidoglycan recognition protein** | | | | | | | | |
| Cluster-19464.0 | 340 | 7.5342↑ | NA | NA | -7.9599↓ | -8.0727↓ | NA | peptidoglycan recognition protein 2 [*Mytilus* *galloprovincialis*] |
| Cluster-6425.35781 | 982 | 1.8863 | 1.9772 | 2.619↑ | 0.43679 | 0.91644 | 0.62347 | peptidoglycan recognition protein 3 [*Mytilus* *galloprovincialis*] |
| Cluster-6425.10745 | 1109 | -4.5197 | -0.093992 | 0.45814 | 4.7918↑ | 5.231↑ | 0.56118 | peptidoglycan recognition protein 1 [*Mytilus* *galloprovincialis*] |
| Cluster-6425.38502 | 1339 | -2.5476 | 0.84374 | -0.031108 | 3.6715↑ | 2.7025↑ | -0.87036 | LysM and putative peptidoglycan-binding domain-containing protein 1 [*Mizuhopecten* *yessoensis*] |
| **Lipopolysaccharide** | | | | | | | | |
| Cluster-6425.23793 | 1106 | -3.8567 | -0.46506 | -1.0669 | 3.68↑ | 2.8685 | -0.62638 | lipopolysaccharide-induced TNF factor 6 [*Mytilus* *galloprovincialis*] |
| Cluster-6425.20202 | 3027 | -1.2221 | 0.87331 | -0.10465 | 2.4275↑ | 1.3774 | -0.98882 | lipopolysaccharide-induced TNF factor 6 [*Mytilus* *galloprovincialis*] |
| Cluster-6425.35616 | 500 | -4.228↓ | 0.21365 | 0.38614 | 4.7744↑ | 4.8535↑ | 0.16597 | lipopolysaccharide-binding protein-like precursor [*Crassostrea* *gigas*] |
| **Bactericidal permeability increasing protein** | | | | | | | | |
| Cluster-6425.35607 | 1255 | -3.5187↓ | 0.4164 | 0.10785 | 4.2578↑ | 3.8329↑ | -0.31674 | bactericidal permeability increasing protein [*Crassostrea* *gigas*] |
| **Interleukin-17** | | | | | | | | |
| Cluster-6425.8575 | 957 | 1.376 | -2.4511↓ | -2.1437↓ | -3.5257↓ | -3.3555↓ | 0.31527 | interleukin-17-5 [*Mytilus* *galloprovincialis*] |
| Cluster-6425.15421 | 803 | 2.8149 | 3.849↑ | 2.8553 | 1.431 | 0.2773 | -0.92775 | interleukin-17-3 [*Mytilus* *galloprovincialis*] |
| **Mytimacin** | | | | | | | | |
| Cluster-24568.1 | 645 | 3.9318↑ | 0.46761 | -1.0823 | -3.1669 | -4.7831 | -1.5592 | Mytimacin-5 [*Mytilus* *galloprovincialis*] |
| Cluster-24568.0 | 596 | 3.5538↑ | 0.8544 | -1.9092 | -2.4027 | -5.231 | -2.7872 | Mytimacin-5 [*Mytilus* *galloprovincialis*] |
| **Defensin** | | | | | | | | |
| Cluster-6425.9412 | 499 | -8.7626↓ | -2.3345↓ | -1.8606 | 6.7987↑ | 7.0619↑ | 0.4706 | RecName: Full=Big defensin; AltName: Full=Defensin; Flags: Precursor |
| **Tumor necrosis factor** | | | | | | | | |
| Cluster-6425.26243 | 3892 | -2.5928↓ | 0.94399 | 0.32385 | 3.879↑ | 3.1459↑ | -0.61483 | PREDICTED: tumor necrosis factor alpha-induced protein 3 isoform X1 [*Crassostrea* *gigas*] |
| Cluster-6425.727 | 1192 | -1.9079 | -2.3544↓ | -0.82533 | -0.080261 | 1.3103 | 1.5286 | PREDICTED: complement C1q tumor necrosis factor-related protein 2 isoform X1 [*Crassostrea* *gigas*] |
| Cluster-6425.47927 | 2015 | 0.71755 | 2.9088↑ | 1.8323 | 2.5207↑ | 1.3575 | -1.0759 | PREDICTED: tumor necrosis factor receptor superfamily member 27 [*Crassostrea* *gigas*] |
| Cluster-6425.38600 | 1606 | -3.3136 | 0.61146 | 0.20716 | 4.242↑ | 3.7379↑ | -0.40814 | tumor necrosis factor ligand superfamily member [*Crassostrea* *gigas*] |
| Cluster-15764.0 | 1176 | -8.1847↓ | -5.509↓ | -4.3816 | 2.9887 | 3.9421 | 1.0995 | PREDICTED: complement C1q tumor necrosis factor-related protein 2-like [*Crassostrea* *gigas*] |
| Cluster-6425.45323 | 2256 | -4.3166 | 2.1219 | 1.0723 | 6.795↑ | 5.693 | -1.0707 | tumor necrosis factor receptor superfamily member 16-like [*Mizuhopecten* *yessoensis*] |
| **Toll-like receptor** | | | | | | | | |
| Cluster-6425.54409 | 1751 | -6.9165↓ | -1.151 | -2.0817↓ | 6.117↑ | 4.9878↑ | -0.93631 | Toll-like receptor 1 [*Argopecten* *irradians*] |
| Cluster-6425.8854 | 2516 | -3.9987↓ | -0.34012 | -1.4393 | 3.9829↑ | 2.7954↑ | -1.0986 | PREDICTED: toll-like receptor 3 isoform X1 [*Crassostrea* gigas] |
| Cluster-6425.52363 | 3191 | -2.8361 | 0.96565 | 0.35049 | 4.1428↑ | 3.477↑ | -0.63086 | toll-like receptor l [*Mytilus* *galloprovincialis*] |
| Cluster-6425.40918 | 2980 | -2.6151↓ | -1.422 | -1.5183 | 1.5109 | 1.3097 | -0.075322 | toll-like receptor c [Myti*l*us *galloprovincialis*] |
| Cluster-6764.3 | 1162 | -5.8573↓ | -0.63025 | -0.31257 | 5.5655↑ | 5.7685↑ | 0.31693 | Toll-like receptor 4 [*Mizuhopecten* *yessoensis*] |
| Cluster-6425.36257 | 2429 | 0.092929 | -2.0751↓ | -2.2998↓ | -1.8433 | -2.1918↓ | -0.23788 | PREDICTED: toll-like receptor 13 [*Crassostrea* *gigas*] |
| Cluster-6425.53511 | 746 | -2.4399 | -1.4194 | -2.0765↓ | 1.3081 | 0.53038 | -0.66143 | toll-like receptor N precursor [*Mytilus* *galloprovincialis*] |
| Cluster-6425.53779 | 1673 | -2.2012 | -0.51875 | -0.77082 | 2.0187↑ | 1.6525 | -0.26764 | toll-like receptor g precursor [*Mytilus* *galloprovincialis*] |
| Cluster-6425.54128 | 654 | -5.1179 | -2.5843↓ | -2.0002 | 2.8496 | 3.363 | 0.58714 | toll-like receptor W precursor [*Mytilus* *galloprovincialis*] |
| Cluster-12548.0 | 2211 | -3.2938 | 2.2986 | -0.47825 | 5.9628↑ | 3.1355 | -2.812 | toll-like receptor O precursor [*Mytilus* *galloprovincialis*] |
| Cluster-6425.3490 | 2428 | -2.7523 | -3.3048↓ | 0.98341 | -0.24797 | 3.8864↑ | 4.3113↑ | PREDICTED: toll-like receptor 6 [*Branchiostoma* *belcheri*] |

**Table S7 Metabolism genes**

| **Transcript no.** | **Transcript length (bp)** | **log2FC M0vsMH** | **log2FC M3vsMH** | **log2FC M9vsMH** | **log2FC M3vsM0** | **log2FC M9vsM0** | **log2FC M9vsM3** | **Nr annotation** |
| --- | --- | --- | --- | --- | --- | --- | --- | --- |
| **Glutamine synthetase** | | | | | | | | |
| Cluster-17359.0 | 1290 | -0.54104 | -1.0368 | 1.3752 | -0.19619 | 2.0424 | 2.3979↑ | glutamine synthetase-like [Crassostrea virginica] |
| **Pyruvate kinase** | | | | | | | | |
| Cluster-6425.13194 | 1181 | 4.4923↑ | 1.147 | 0.82397 | -3.0356↓ | -3.5211↓ | -0.30823 | PREDICTED: pyruvate kinase isoform X1 [Drosophila rhopaloa] |
| **Malate synthase** | | | | | | | | |
| Cluster-6425.15105 | 2900 | -2.7955 | 0.0093883 | 0.2981 | 3.1164↑ | 3.3277↑ | 0.28943 | Malate synthase [Mizuhopecten yessoensis] |
| **Transaldolase** | | | | | | | | |
| Cluster-12784.0 | 1720 | -7.2618 | -7.4463↓ | -7.4003↓ | 0.093541 | 0.068231 | 0.083511 | transaldolase [Acanthamoeba castellanii str. Neff] |
| **Fructose-bisphosphate aldolase** | | | | | | | | |
| Cluster-6425.25500 | 1727 | -2.2302↓ | -1.6058 | -1.0331 | 0.98066 | 1.4095 | 0.57083 | PREDICTED: fructose-bisphosphate aldolase [Crassostrea gigas] |
| **Phospho-2-dehydro-3-deoxyheptonate aldolase** | | | | | | | | |
| Cluster-275.0 | 843 | -4.6381 | -6.6241↓ | -6.6043↓ | NA | NA | NA | PREDICTED: putative phospho-2-dehydro-3-deoxyheptonate aldolase [Plutella xylostella] |
| **Hexokinase** | | | | | | | | |
| Cluster-6425.12523 | 1760 | 2.3562 | 3.7958↑ | 3.0158↑ | 1.7741 | 0.84177 | -0.7038 | hexokinase-1-like [Crassostrea virginica] |
| Cluster-6425.32607 | 2364 | -2.5526↓ | -1.6403 | -1.055 | 1.2518 | 1.6892 | 0.60588 | Hexokinase type 2 [Mizuhopecten yessoensis] |
| Cluster-6425.43549 | 1386 | NA | 5.8107↑ | 5.4424↑ | 4.8677↑ | 4.4169↑ | -0.34428 | hexokinase [Crassostrea angulata] |
